# Monomeric C-Reactive Protein in Serum With Markedly Elevated CRP Levels Shares Common Calcium-Dependent Ligand Binding Properties With an *in vitro* Dissociated Form of C-Reactive Protein

**DOI:** 10.3389/fimmu.2020.00115

**Published:** 2020-02-04

**Authors:** Robert D. Williams, Jennifer A. Moran, Anthony A. Fryer, Jamie R. Littlejohn, Harry M. Williams, Trevor J. Greenhough, Annette K. Shrive

**Affiliations:** ^1^School of Life Sciences, Keele University, Staffordshire, United Kingdom; ^2^Department of Clinical Biochemistry, Institute for Applied Clinical Sciences, University Hospitals of North Midlands, Keele University, Staffordshire, United Kingdom

**Keywords:** monomeric C-reactive protein, serum C-reactive protein, dissociated C-reactive protein, calcium-dependent ligand binding, phosphocholine, C-polysaccharide, pentraxin, innate immunity

## Abstract

A monomeric form of C-reactive protein (CRP) which precipitates with cell wall pneumococcal C polysaccharide (CWPS) and retains the ability to reversibly bind to its ligand phosphocholine has been produced through urea-induced dissociation at an optimized concentration of 3 M urea over a 10 weeks period. Dissociated samples were purified via size exclusion chromatography and characterized by western blot, phosphocholine affinity chromatography and CWPS precipitation. Human serum samples from patients with raised CRP levels (>100 mg/L as determined by the clinical laboratory assay) were purified by affinity and size exclusion chromatography and analyzed (*n* = 40) to determine whether circulating monomeric CRP could be detected *ex vivo*. All 40 samples tested positive for pentameric CRP via western blot and enzyme linked immunosorbent assay (ELISA) analysis. Monomeric C-reactive protein was also identified in all 40 patient samples tested, with an average level recorded of 1.03 mg/L (SE = ±0.11). Both the *in vitro* monomeric C-reactive protein and the human serum monomeric protein displayed a molecular weight of approximately 23 kDa, both were recognized by the same anti-CRP monoclonal antibody and both reversibly bound to phosphocholine in a calcium-dependent manner. In common with native pentameric CRP, the *in vitro* mCRP precipitated with CWPS. These overlapping characteristics suggest that a physiologically relevant, near-native monomeric CRP, which retains the structure and binding properties of native CRP subunits, has been produced through *in vitro* dissociation of pentameric CRP and also isolated from serum with markedly elevated CRP levels. This provides a clear route toward the in-depth study of the structure and function of physiological monomeric CRP.

## Introduction

C-reactive protein (CRP) belongs to the family of proteins known as the pentraxins, which have been highly conserved throughout evolution and can be identified in species ranging from mammals and fish to the primitive Atlantic horseshoe crab, *Limulus polyphemus* (1). The human form of CRP displays a ring structure of five non-covalently bound subunits around a central pore, with each subunit containing 206 amino acids folded into two antiparallel β-sheets with a flattened jellyroll topology (2). Two calcium ions are contained within each promoter, forming the site for the binding of ligands such as phosphocholine (PCho) expressed on the surface structures of pathogens and dead or damaged cell membranes (2, 3). The primary role of CRP within the human body is believed to be the initiation of the innate immune response via the classical complement pathway, cytokine induced inflammation and interactions with antibodies. However, more complex roles for CRP have been described, including immune exacerbation, regulation and suppression (4–7).

In normal, healthy individuals, baseline levels of CRP range from 1 to 5 mg/L (8). During infection, inflammation or tissue damage, CRP levels will rise acutely to elicit a sufficient immune response over a 24–72 h period. Due to this rapid increase in serum levels of CRP, it is routinely measured as a clinical biomarker for infection or inflammation in the healthcare setting. Over recent years, evidence has emerged linking CRP to the development and progression of chronic, auto-inflammatory diseases such as cardiovascular disease (9, 10). It has been hypothesized that CRP exerts an alternative, pro-inflammatory role in disease development, with *in vitro* studies demonstrating up-regulation of cell adhesion molecules, activation of endothelial cells, deposition within atherosclerotic lesions and the increased production of inflammatory cytokines; all common features found in the pathophysiology of cardiovascular disease (8, 11–15). This evidence has led to the development of a high sensitivity CRP assay which can accurately measure CRP levels <3 mg/L and assess an individual's risk of developing cardiovascular disease (9, 10).

Although human pentameric CRP (pCRP) is extremely stable under physiological conditions, there is increasing evidence which suggests a biological role for a monomeric form of CRP, commonly denoted mCRP (16–19). A wide variety of *in vitro* and *ex vivo* monomeric CRP (mCRP) conformers have been reported, ranging from the *in vitro* denatured mCRP produced by high concentrations of urea (up to 8 M) (20) through reduced membrane bound mCRP (21) to the hybrid mCRP with near native structure (22). Monoclonal antibodies for the various conformers have been generated, including those targeting neo-epitopes revealed by pCRP subunit dissociation, and used to identify monomeric forms as distinct from pCRP (23–25). The urea dissociated mCRP of Potempa et al. (20) remains very much the prototypical mCRP, being used in a wide variety of studies for over 30 years. This mCRP displays a decrease in solubility (being insoluble in calcium concentrations as low as 0.05 mM) and isoelectric point and a loss of calcium-dependent binding to the prototypical CRP ligand C-polysaccharide (20). This monomer could not be re-associated back into its pentameric form, the dissociation being accompanied by significant changes in both structure and function (26). On the basis of reduction of the intrachain disulphide in this mCRP, but not in pCRP, by reducing agents such as dithiothreitol, several reports have shown discrimination between pentameric and monomeric CRP (21, 27, 28).

Several models suggesting a physiological mechanism for the formation of mCRP have been postulated within the literature. The most widely accepted involves dissociation through calcium-dependent binding of pCRP to cell membranes (17, 18, 25, 27–32) although a clear picture is yet to emerge of the nature and fate of the resulting mCRP conformer. Binding to microvesicles has been proposed to lead to pCRP structural changes that allow binding of C1q and hence complement activation (27). In addition, increasing experimental evidence within the literature suggests a pro-inflammatory role for mCRP (33) and a link between mCRP and the progression of certain auto-inflammatory diseases within the body. Similarly to that described previously for pCRP, mCRP displays an ability to stimulate cytokine production and up-regulate the production of adhesion molecules as well as induce thrombus growth formation and alter fibrin properties and tissue factor expression (18, 34). It has been hypothesized that circulating micro-particles, generated from lipid membranes, can not only induce pCRP dissociation, but also act as a mode of transport around the body to sites of intensified inflammation (30). The failure of standard clinical CRP tests, employed in a large proportion of healthcare laboratories, to detect mCRP that is bound to circulating micro-particles has also been highlighted (35).

More recently, the development of ELISA methods which can quantify mCRP in clinical serum and tissue samples down to 1 ng/ml have been reported (24, 36). Zhang et al. (36) demonstrated a sandwich ELISA that detected mCRP in patients with skin-related autoimmune disorders, with a statistical difference in mCRP levels when compared to the control group, even when native, pentameric samples showed no such difference (35). Levels of serum mCRP reported by Zhang et al. (36) were in the ng/ml range, as were those reported by Wang et al. (24) who employed a monoclonal antibody based on the C-terminal octapeptide of CRP, proposed to be exposed on pCRP dissociation by treatment with 8 M urea, in a study of mCRP levels in acute myocardial infarction (AMI). The antibody was shown to recognize mCRP from denatured pCRP, but not pCRP itself. This study of patients (*n* = 101) who had recently suffered with an AMI reported an average recorded mCRP serum level of 0.021 mg/L (24), with six patients out of 42 with raised levels of pCRP (3–285 mg/L) displaying detectable mCRP levels of 0.0057 mg/L−0.1227 mg/L.

Our aim was to generate, and find in serum with markedly elevated CRP levels, a near native mCRP that retains as far as possible the structure and binding of the monomer in the pentameric molecule. We describe the production, purification and characterization of *in vitro* mCRP, produced under milder dissociation conditions than those usually employed, alongside the analysis of human serum samples from patients exhibiting raised CRP levels (>100 mg/L) by clinical assay. This analysis reveals an *ex vivo* form of mCRP in all patient samples analyzed. Both forms of mCRP were recognized by the same anti-CRP monoclonal antibody, both were soluble in tris buffered saline with 5–10 mM CaCl_2_, and both reversibly bound to PCho in a calcium-dependent manner.

## Materials and Methods

Standard buffers used were as follows: buffer A: (storage buffer) 20 mM tris(hydroxymethyl) aminomethane (tris), 280 mM NaCl, 5 mM CaCl_2_, 0.01% sodium azide, pH 7.4; buffer B: (elution buffer) 50 mM tris, 150 mM NaCl, pH 7.2; buffer C: (equilibration buffer) 10 mM CaCl_2_, 50 mM tris pH 7.4, 150 mM NaCl, 0.01% sodium azide; buffer D: (chelating buffer) 10 mM ethylenediaminetetraacetic acid (EDTA), 50 mM tris pH 7.4, 150 mM NaCl, 0.01% sodium azide.

### ELISA Protocol

The general procedures for ELISA were as follows: All ELISA analysis was carried out on a sterile, 96-well plate (Costar). 50 μl of each sample was added to each well. Plates were blocked with 3% bovine serum albumin (BSA) and washed with phosphate buffered saline (PBS)-Tween (Sigma). The primary antibody used was a monoclonal anti-C-reactive protein antibody (CRP-8, Sigma) produced in a mouse; the secondary antibody was an anti-mouse IgG (whole molecule)–peroxidase antibody produced in rabbit (Sigma). Both were used at 1:40,000 dilution. Positive and negative controls were included in analysis and all samples were tested in triplicate. The developing substrate used was 3, 3′, 5, 5′, tetramethylbenzidine (TMB) liquid substrate (Sigma). ELISA plates were left to develop for 10 min prior to addition of 2 M sulfuric acid. Plates were read at 450 nm on a BioTek EL800 plate reader using Gen5 software.

### Production of *in vitro* Monomeric C-Reactive Protein

Native pentameric human CRP (pCRP) was purchased from Scripps in storage buffer (buffer A). Dissociation was achieved through the addition of pCRP to a range of dissociation buffers (20 mM tris, 280 mM NaCl, 1 mM EDTA, and 2, 2.5, 3, and 4 M urea), followed by storage at 4°C for periods of up to 12 weeks.

### Phosphocholine (PCho) Affinity Chromatography

Immobilized *p-*aminophenyl phosphoryl choline (APC) was purchased from Fisher Scientific in the form of a gel, cross-linked with 6% beaded agarose. The binding capacity of the APC was >3 mg of CRP per 1 ml of gel. All affinity chromatography experiments for both *in vitro* and *ex vivo* CRP were run using the Biologic LP Chromatography System (BioRad) with a flow rate of 0.5 ml/min throughout. The absorbance was measured at 280 nm. Prior to samples being run through the column, the column was equilibrated with 20 column volumes of equilibration buffer (buffer C) and the UV absorbance baseline reading was then blanked to a value of 0 absorbance units (Au) prior to application of the sample. Once the entire sample had been applied, the column was re-equilibrated with 5 column volumes of buffer C followed by elution with the chelating buffer (buffer D) until the UV absorbance reading had returned to its baseline value of 0 Au.

### Purification of *in vitro* Monomeric C-Reactive Protein (mCRP)

mCRP and pCRP in the urea dissociated samples were separated and purified using the AKTA explorer 100 Fast Protein Liquid Chromatography (FPLC) system (GE Healthcare) with a HiLoad 16/60 Superdex 200 pg column. Calibration was performed using a Gel Filtration Calibration Kit (GE Healthcare), with the included molecular weight markers ferritin, aldolase, conalbumin, ovalbumin, and ribonuclease (elution buffer: buffer B). The void volume was determined using Blue Dextran (Sigma). Samples were injected onto the column via a 2 ml sample injection loop and elution was monitored by UV absorbance at 280 nm at a flow rate of 0.5 ml/min. Samples were eluted off and collected in 5 ml fractions in the storage buffer A.

### SDS PAGE and Western Blots of Purified pCRP and *in vitro* mCRP Samples

Both purified dissociated CRP samples and CRP purified from human serum were confirmed to contain CRP through sodium dodecyl sulfate polyacrylamide gel electrophoresis (SDS PAGE) and western blot analysis. Samples were run under non-reducing conditions on a 12.5% resolving and 4% stacking gel. All reagents were purchased from Sigma. SDS PAGE was run in a BioRad mini-Protein II cell at 200 V for approximately 45–50 min. All samples were run with positive and negative controls and molecular weight markers.

Western blot analysis was performed using the Mini Trans-Blot Electrophoretic Transfer Cell (BioRad), using nitrocellulose blotting paper or Polyvinylidene difluoride (PVDF) membrane. The transfer was run at 350 mA for 1 h at 4°C. Membranes were blocked with Marvel dried milk powder and washed with BLOTTO (Marvel dried milk, Triton-X 100 or Tween-20, and tris-buffered saline). The primary antibody, a monoclonal anti-C-reactive protein antibody (CRP-8, Sigma) produced in a mouse, was used at a 1:1,000 dilution except for the mCRP purified from human serum (1:400) and for the non-dissociated pCRP following treatment with urea (1:200). The secondary antibody, an anti-mouse IgG (whole molecule)–peroxidase antibody produced in rabbit (Sigma), was used at a 1:40,000 dilution except for the non-dissociated pCRP following treatment with urea (1:2,000). Clarity Western Enzyme Chemiluminescence substrate (BioRad) or Thermo Scientific™ SuperSignal™ West Pico Chemiluminescent Substrate diluted x4 was used to detect protein bands which were visualized using a CCD digital image detector. Exposure time was approximately 30–60 min.

### Binding of *in vitro* mCRP to Phosphocholine

The calcium-dependent binding to PCho of the size exclusion purified mCRP from the 3 M urea dissociation was assessed using PCho affinity chromatography as described above. Protein samples were prepared by diluting 500 μl (250 μg) of the mCRP in 5 ml of buffer C. Larger volumes of samples were used to prevent air bubbles entering the column whilst applying a sample.

### Precipitation Studies With *Streptococcus pneumoniae* Cell Wall Polysaccharide

#### ELISA Calibration

50 μl of calcium buffer (buffer A) was added to columns 1–12, rows A–F of the ELISA plate. 50 μl (3.0 μg in 50 μl, 60 mg/L) of pCRP (Scripps) was added to column 1, rows A–C, and mCRP (*in vitro*, purified following the 3 M dissociation) to rows D–F. Serial dilution through to column 11, rows A–F was carried out leaving buffer A in column 12 rows A–F (negative control).

#### CRP/CWPS Precipitation

Native pCRP (Scripps) alongside mCRP (dissociated with 3 M urea and purified by size exclusion), were incubated with varying amounts of *Streptococcus pneumoniae* cell wall C-polysaccharide (CWPS; Oxford Biosystems). CWPS quantities used were 0.1, 0.5, 1, 10, 20, 50, 100, 200, 300 μg. Both CRP samples contained 20 μg in 400 μl buffer A. All samples were incubated at 37°C for 1 h whilst being gently agitated. The samples were then centrifuged at 6,500 × g for 15 min to pellet the precipitate and the supernatant from each sample was transferred to separate tubes. Precipitation levels were calculated based on the remaining concentration of protein within the supernatant via ELISA following the general procedures outlined above. CRP concentrations were determined using the calibration line equations and the total level of precipitation was calculated by subtracting the total amount of CRP remaining in solution post CWPS incubation from the initial amount (20 μg).

### Protein Concentration and Quantification

Eluted protein samples were stored for use at 4°C in the various elution buffers following concentration using a Vivaspin Centrifugal Concentrator (Fisher Scientific), 10 kDa molecular weight cut off. The concentrator was initially primed with deionised water and spun at 1,000 x g, 4°C for 10 min. The protein sample was then spun at 1,000 × g, 4°C for 15 min or until the desired concentration had been achieved. The protein content of the CRP samples was determined from absorbance at UV 280 nm. The extinction coefficient was calculated from the CRP sequence as 1.7 (Expasy, http://web.expasy.org/protparam/).

### Ethical Application and Research Study Design

Ethical approval for the human serum samples pilot study was granted by UK National Health Service Research Ethics Service (NRES) Committee South Central—Oxford B (REC reference: 15/SC/0179). A total of 40 inpatients at the University Hospitals of North Midlands (UHNM), identified only by sample numbers, were recruited who were over the age of 18, were able to give informed consent, were due to undergo a routine blood test and who had a serum CRP level >100 mg/L as measured by immunoturbidimetry on the Siemens Advia 2400 assay (Siemens Healthcare Diagnostics Limited, Frimley, UK). All aspects of our study were compliant with the regulatory terms and conditions set out by the NRES committee and Keele University local ethics committee.

### Human Serum Sample Collection

Whole human blood was collected into BD Vacutainer SST gel tubes without anticoagulant. Sample tubes were left for 30 min to clot and centrifuged at 3,500 g for 7 min to separate the serum. Serum samples were stored at 4°C.

### Purification of CRP From the Human Serum Samples

CRP was purified from human serum with markedly elevated CRP levels (>100 mg/L), as determined by the routinely used clinical assay, via PCho affinity chromatography as described above. Serum sample volumes were 900 μl. Eluted samples were then purified via size exclusion chromatography as described for the *in vitro* analysis to separate out potential CRP isoforms. Briefly, samples were applied to the column via a 2 ml sample injection loop. Sample elution using buffer A was monitored by UV absorbance at 280 nm at a flow rate of 0.5 ml/min. pCRP and mCRP elution times were calculated prior to purification based on the *in vitro* studies.

### ELISA Analysis of CRP From Human Serum Samples

Following elution from the size exclusion chromatography column, the purified pCRP and mCRP from the human serum samples were also subject to ELISA analysis following the general procedures outlined above. The purified human serum pCRP and mCRP fractions were diluted in buffer A 1:100 and 1:10, respectively, prior to analysis. For the calibration, 50 μl of calcium buffer (buffer A) was added to columns 1–8, rows A–B of the ELISA plate. 50 μl of either native pCRP (Scripps) or mCRP (*in vitro*, purified following the 3 M urea dissociation) was added to column 1, rows A–B (0.165 μg, 0.0033 mg/ml). Serial dilution through to column 7, rows A–B was carried out leaving only calcium buffer in column 8, rows A–B (negative control).

### Experimental Procedures for the Human Serum Samples Applied to Commercially Sourced CRP

The general methods for affinity and size exclusion chromatography, and for ELISA, are given elsewhere. Specifics for this experimental procedure were as follows: A total of 3.0 mg (1.2 ml at 2.5 mg/ml) CRP (Binding Site) was applied to the PCho affinity column. EDTA eluted 3 ml CRP fractions were collected and concentrated using a Vivaspin Turbo 15 (10 kDa MWCO) at 4°C, 2,100 rpm for 10 min. Following recalibration of the size exclusion column (necessary due to a change of column) using molecular weight standards CRP (115 kDa), conalbumin (75 kDa), ovalbumin (43 kDa), carbonic anhydrase (29 kDa, ribonuclease (13.7 kDa), and aprotinin (6.5 kDa), size exclusion FPLC of this PCho purified CRP was carried out at a flow rate of 0.5 ml/min. Two size exclusion runs were carried out, the first loading 500 μg of the CRP, in excess of the CRP in the serum sample with the largest clinically assayed CRP level, the second loading 100 μg. The fractions spanning the mCRP elution volume indicated by the calibration were collected (six 5 ml fractions in each case), diluted 1:100 and 1:50 in calcium buffer and subjected to ELISA (primary antibody CRP-8) in triplicate for all fractions. Two ELISA plates were completed, one for the 500 μg size exclusion chromatography (SEC) fractions, the other for the fractions from the 100 μg SEC experiment. Both included a calibration in triplicate using serial dilution of 6 ng of mCRP (3 M urea dissociated) along with positive (4 ng mCRP) and negative (calcium buffer, buffer A) controls.

## Results

### Production and Purification of *in vitro* Monomeric C-Reactive Protein

**Figure 1:**
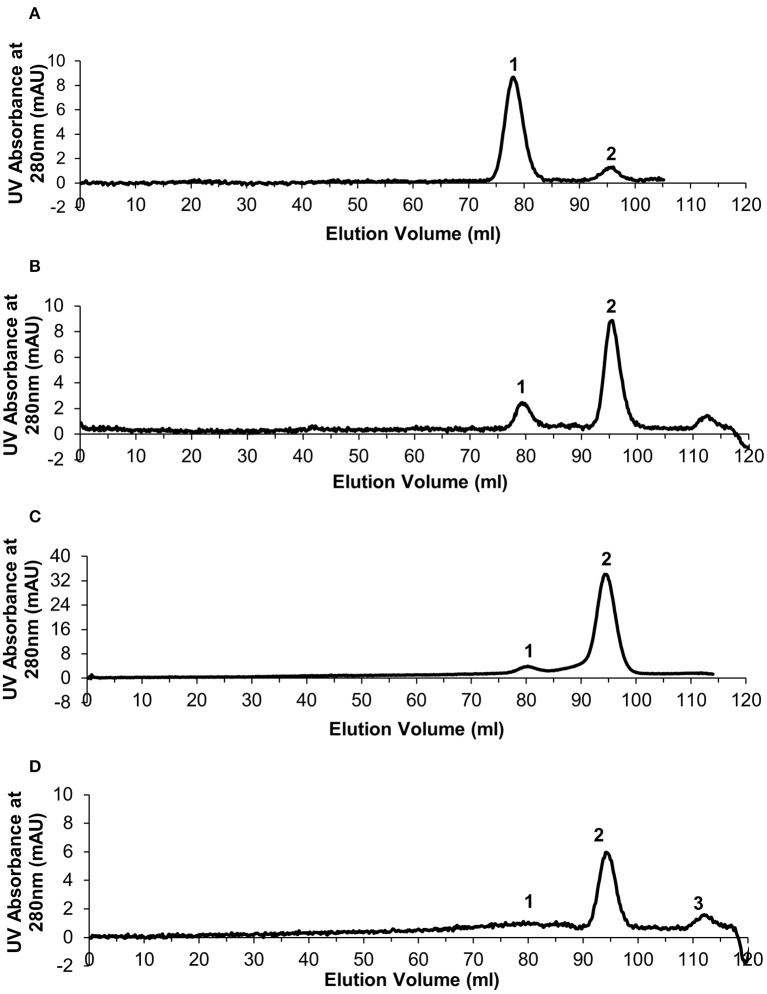
Generation of monomeric CRP. Size exclusion chromatography traces for the urea dissociation experiments with 1 mM EDTA. In each case Peak 1 represents eluted pentameric CRP and peak 2 represents eluted monomeric CRP. **(A)** 2 M urea **(B)** 2.5 M urea **(C)** 3 M urea **(D)** 4 M urea—peak 3 determined as not significant based on calculated molecular weight.

Size exclusion chromatography was used to assess the level of dissociation of pentameric CRP into monomeric CRP in the presence of 1 mM EDTA under a range of urea concentrations, each over a period of several weeks, and to purify out both pCRP and mCRP from the sample. This resulted in the selection of 2–4 M urea over a period of 10 weeks. For the calibration, the void volume was calculated to be 46.79 ml and the column volume 120.6 ml. Elution volumes for the molecular weight standards are given in Table S1 and the calibration graph in Figure S1. The elution traces are shown in Figure 1 and the elution volumes and calculated molecular weights in Table 1. There is no evidence in the elution traces of intermediate CRP aggregations comprising 2, 3, or 4 subunits. 5 ml fractions were collected throughout the elutions, the two fractions associated with each peak, for example 75–80 ml, 80–85 ml for pCRP, 90–95 ml, 95–100 ml for mCRP for the 3 M dissociation, being collected, pooled, and concentrated in the elution buffer for further characterization. Measurement of protein concentration (absorption at UV 280 nm) for the concentrated pCRP and mCRP samples suggested losses of up to 50% with respect to the starting pCRP. Losses do vary significantly, with a direct correlation between the amounts of CRP, and there appears to be a “baseline” loss, particularly during the concentration step, irrespective of the quantity. This clearly has a much greater effect for small quantities. For quantities of CRP in the mg range yields of up to 90% following size exclusion and concentration are achievable, while for small quantities, in the μg range, losses of up to 50% are common.

**Table 1:** Size exclusion column elution volumes and calculated molecular weights following pCRP dissociation via varying concentrations of urea.

	**2 M urea**		**2.5 M urea**		**3 M urea**		**4 M urea**	
	**Elution vol (ml)**	**MW(kDa)**	**Elution vol (ml)**	**MW(kDa)**	**Elution vol (ml)**	**MW(kDa)**	**Elution vol (ml)**	**MW(kDa)**
pCRP	79.02	108	79.09	109	79.60	103	79.27	107
mCRP	95.65	21.3	95.68	21.2	94.97	22.0	94.90	22.0

The 3 M urea dissociation (Figure 1C) clearly maximizes both mCRP yield and pCRP to mCRP conversion and was therefore used in subsequent studies. No evidence of dissociation was observed in the presence of calcium (5 mM) (results not shown).

### Western Blots of the Purified *in vitro* pCRP Dissociation Products

**Figure 2:**
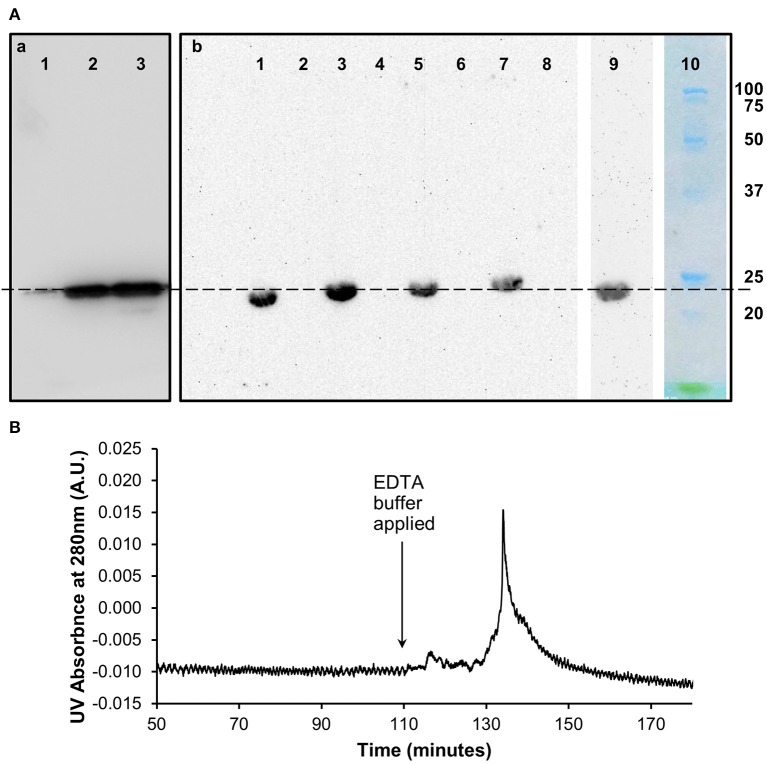
Characterization of *in vitro* monomeric CRP. **(A)** Western blots (non-reducing 12.5% SDS) of the (a) pentameric and (b) monomeric fractions of CRP from the size exclusion chromatography analysis following dissociation with urea. (a) Lane 1 (0.1 μg) and lane 2 (1 μg) contain the pentameric fraction separated from the 3 M urea dissociated sample with control pCRP in lane 3. (b) Lanes 1, 3, 5, 7 contain the monomeric fraction separated from the 2, 2.5, 3, and 4 M urea dissociated samples, respectively. Lanes 2, 4, 6, 8 contain buffer. Lane 9: 3 M urea dissociated mCRP (Lane 5) subsequently eluted from the PC affinity column by EDTA (b). This Lane was excised from the blot after 5 min exposure, the remaining Lanes 1–8 being exposed for a further 40 min. Lane 10 Molecular weight markers (Biorad precision plus unstained) excised before blotting and stained with Coomassie brilliant blue. The dashed line indicates 23 kDa. **(B)** PCho affinity chromatography of the size-exclusion purified, *in vitro* produced mCRP (from 3 M urea, Lane 5, Ab). The major peak represents elution of mCRP by the calcium chelator EDTA. Time represents elapsed time after the mCRP sample was loaded. The Western blot of the PCho affinity purified mCRP is shown in Lane 9 of the blot in (Ab).

Western blots (Figure 2) of the size exclusion chromatography eluted monomeric and un-dissociated pentameric CRP fractions (Figure 1) confirmed the ability of the proteins to still be recognized by the anti-CRP antibody.

### Binding of Purified *in vitro* Monomeric CRP to Phosphocholine

The 3 M urea dissociated mCRP purified by size-exclusion was applied to a PCho affinity column followed by elution with EDTA chelating buffer (buffer D) (Figure 2). The pooled mCRP fractions were concentrated and subjected to western blot analysis (Figure 2) confirming the ability of the protein to still be recognized by the anti-CRP antibody. Measurement of protein concentration (absorption at UV 280 nm) for the concentrated mCRP sample suggested losses of up to 70% with respect to the starting mCRP.

### Precipitation Studies With *Streptococcus pneumoniae* Cell Wall Polysaccharide

**Figure 3:**
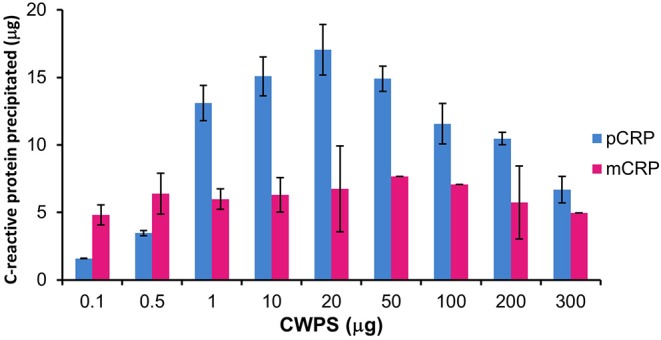
Precipitation of pCRP (20 μg) and mCRP (3 M urea, 20 μg) with CWPS. Errors shown are ± standard deviation (*n* = 2).

Precipitation of mCRP and pCRP with CWPS was carried out. The ELISA calibration data and graphs for pCRP and mCRP are given in Tables S2, S3, Figures S2, S3. The levels of precipitation for both native pCRP and *in vitro* produced, size exclusion purified mCRP (3 M urea dissociated) when incubated with varying amounts of CWPS are shown in Figure 3.

Pentameric CRP displays the classic precipitation curve (37) with little precipitation with CWPS at either end of the curve where protein or ligand is in significant excess. Approximately 2 μg of pCRP precipitates out of solution when incubated with 0.1 μg of CWPS, while 7 μg of pCRP precipitates out of solution when incubated with 300 μg of CWPS. Maximum precipitation (17 μg out of a total of 20 μg of pCRP) occurs where protein and CWPS quantities are the same (20 μg). Precipitation by mCRP maintains a general level of approximately 5–7 μg irrespective of the amount of CWPS, peaking at just over 7 μg, when mCRP is incubated with 50 μg of CWPS, although this peak level is still markedly lower than that for pCRP with 50 μg of CWPS (approximately 15 μg).

### Purification of CRP From Human Serum Samples

**Figure 4:**
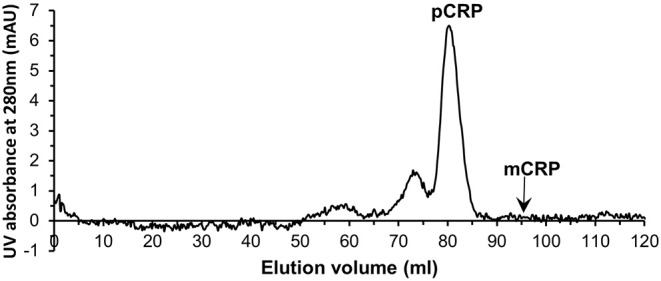
Typical size exclusion chromatography trace of one of the PCho affinity purified human serum samples. The pCRP peak is clear while no peak is visible for mCRP.

Serum samples from 40 patients with elevated CRP levels (>100 mg/L by clinical assay) were purified individually by PCho affinity chromatography (Figure S4). Further purification and characterization then followed the procedures employed for the *in vitro* mCRP. All affinity purified samples were subjected to size exclusion chromatography (Figure 4). The major peak eluted at 79.01 ml (110 kDa; Figure S1; pCRP]. Although no mCRP peak, expected at 95 ml, was observed in any of the purified samples (Figure 4), fractions 90–95 and 95–100 ml (see Table 1, Figure 1) were pooled for further analysis.

### Analysis of Pentameric and Monomeric C-Reactive Protein Within the Human Serum Samples

**Figure 5:**
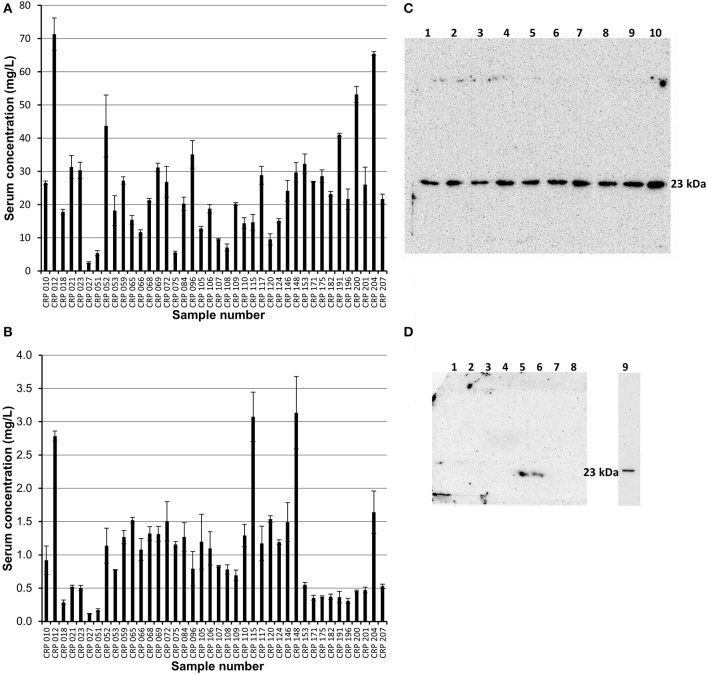
ELISA and western blot analysis of pentameric and monomeric CRP in the human serum samples. **(A,B)** ELISA analysis. All 40 samples tested positive for both pCRP and mCRP. Scores are based on the average value minus the average control. Errors shown are ± standard deviation (*n* = 3). **(A)** Samples were diluted 1:100 in triplicate. The average concentration of pCRP is 23.3 mg/L (*SE* = ±2.3). **(B)** Samples were diluted 1:10 in triplicate. The average concentration of mCRP is 1.03 mg/L (*SE*= ± 0.11). **(C,D)** Western blot (non-reducing 12.5% SDS) analysis of the pentameric and monomeric human serum CRP fractions eluted from the size exclusion column. **(C)** Pentameric CRP was positive for all 40 samples tested (CRP 148, 146, 124, 120, 117, 115, 110, 109, 108 in lanes 1–9, respectively, are shown here). Control pCRP (~23 kDa) sample in lane 10. **(D)** Monomeric CRP: eight of the forty serum samples (018, 021, 023, 109, 115, 148, 153, 175) are shown with two samples (Lane 5, sample 115; Lane 6, sample 148; the two highest values in the mCRP ELISA) positive for mCRP at a molecular weight of approximately 23 kDa. Lane 9 contains urea dissociated mCRP control at 23 kDa, this lane being excised from the gel after 2.5 min with the remainder exposed for a further 42.5 min. One other sample (204, the fourth highest ELISA mCRP value) was positive for mCRP in western blots (data not shown). The mCRP from sample 012, which gave the highest pCRP ELISA reading and the third highest mCRP ELISA value, could not be visualized by western blotting.

Affinity chromatography followed by size exclusion purified fractions of both pentameric and monomeric CRP were each subject to ELISA analysis and quantification. The calibration details are given in Table S4, Figure S5 (mCRP) and Table S5, Figure S6 (pCRP). All CRP concentrations were adjusted to represent the concentration in the initial serum samples. All 40 purified pCRP samples tested positive in the ELISA for the presence of CRP (Figure 5). The lowest recorded value of pentameric CRP for any of the samples was calculated at 2.5 mg/L, with the highest being recorded at 71.3 mg/L and a mean value of 23.3 mg/L (SE = ±2.3). All 40 samples of the purified monomeric form of CRP from serum also tested positive for the presence of CRP (Figure 5) with a mean value of 1.03 mg/L (SE = ±0.11), the highest value being 3.14 mg/L (sample 148). There is no significant correlation between the serum mCRP and pCRP (Figure 5) concentrations across all the samples with, for example, the higher mCRP values being associated in equal measure with both high and low pCRP levels (Figure S7). The purified pentameric and monomeric CRP fractions from the size exclusion column were also subject to western blot analysis. All 40 pCRP samples tested positive for CRP, a representative selection is shown in Figure 5. For mCRP, the western blots revealed CRP in 3 out of the 40 patient sample derived mCRP samples (Figure 5).

### Experimental Procedures for the Human Serum Samples Applied to Commercially Sourced CRP

**Figure 6:**
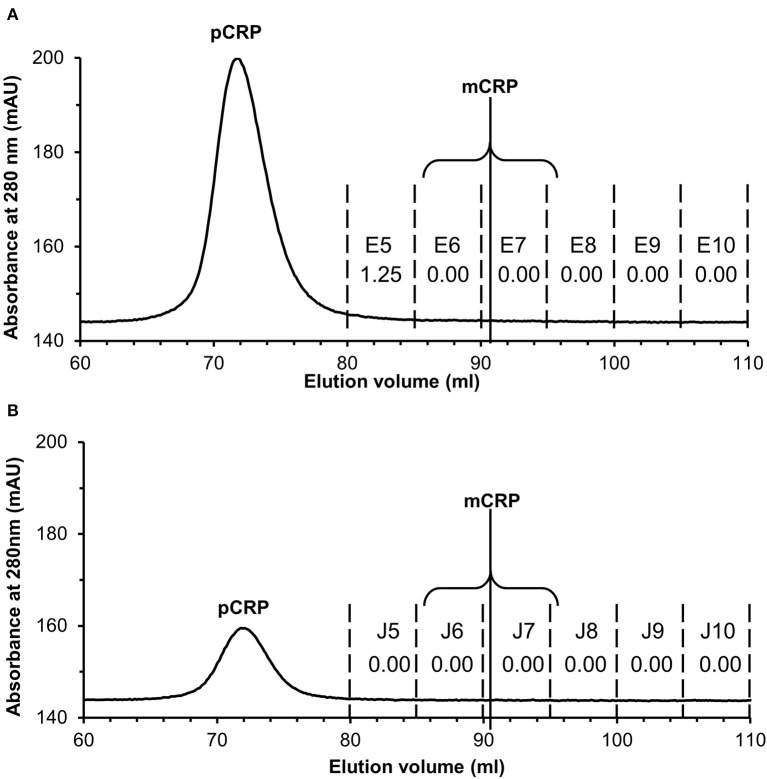
Size exclusion FPLC of PCho re-purified CRP. Elution traces are shown for both **(A)** 500 μg and **(B)** 100 μg CRP loaded onto the column. Fractions E5–E10 and J5–J10 (5 ml each) were diluted 1:100 and 1:50 in calcium buffer and subjected to ELISA (primary antibody CRP-8) in triplicate (see Tables S7–S10, Figures S10, S11). Total CRP in mg/L averaged over the two dilutions is shown for each fraction. Fraction E5 for the higher (500 μg) loading shows a positive reading associated with the pCRP tail, while CRP could not be identified in any other fraction or in any fraction of the 100 μg run. The two fractions within which mCRP would elute are indicated.

Application to commercially sourced CRP of our experimental procedures (PCho-affinity chromatography followed by separation of pCRP and mCRP by SEC) for isolation of mCRP from patient serum samples, all showing markedly elevated CRP levels >100 mg/L by clinical assay, was carried out to confirm that these purification procedures do not produce the mCRP detected in patient samples. PCho affinity chromatography of the commercially sourced CRP gave a clean, sharp peak with EDTA elution (Figure S8A). EDTA eluted 3 ml CRP fractions G9–G11 (Figure S8A) were collected and concentrated to 2 ml at 1.44 mg/ml (2.88 mg CRP). Application of the PCho purified CRP to the size exclusion column (two separate runs, loading 500 μg and 100 μg) showed the pCRP peak at the expected position of 70–71 ml (Figures S8B,C) indicated by the calibration (Table S6, Figure S9) with no discernible peak at the mCRP position (90–91 ml). ELISA analysis of the PCho + size exclusion purified native CRP for both the 500 μg (Tables S7, S8, Figure S10) and 100 μg (Tables S9, S10, Figure S11) size exclusion runs revealed no detectable mCRP. Fraction E5 for the higher (500 μg) size exclusion column loading shows a positive CRP reading of 1.25 mg/L associated with the pCRP tail (Figure 6A), while CRP could not be identified in any fraction of either run in the region of the mCRP elution volume. (Figure 6B).

## Discussion

C-reactive protein dissociation experiments using commercially sourced pCRP and a range of urea concentrations were carried out over a 10 week period to establish a suitable *in vitro* protocol for the production of monomeric C-reactive protein. The aim was to minimize the strength of urea whilst still inducing a high level of dissociation over a realistic time frame. Although the mechanism of dissociation in the presence of urea is not clear, we believe this is the most suitable method of those reported in the literature (20) since attempts to treat pentameric CRP with heat or acidic conditions resulted in complete denaturation of the protein (data not shown). The most appropriate urea concentration for the almost complete dissociation of pentameric to monomeric CRP (>90%) was 3 M (Figure 1). Lower concentrations of urea resulted in partial or no dissociation, while the removal of the CRP calcium ions was shown to be crucial regardless of the urea concentration. Dissociated samples were successfully separated into mCRP and residual pCRP by size exclusion chromatography, western blot analysis confirming the ability of both proteins to still be recognized by the anti-CRP antibody.

Further characterization of the *in vitro* mCRP was carried out using PCho affinity chromatography and precipitation with CWPS. The PCho affinity chromatography mCRP peak (Figure 2) confirms the calcium-dependent PCho-binding ability of the *in vitro* produced mCRP, further verified by western blot analysis of the eluted peak which, as expected, was positive for CRP. Based on this experimental evidence it was concluded that the urea dissociation experiments successfully dissociated pCRP into its constituent subunits yielding an *in vitro* form of mCRP that retained its PCho-binding ability. Precipitation studies with CWPS (Figure 3) revealed that the *in vitro* mCRP produced here also retains the ability to recognize its prototypical ligand CWPS. However, unlike the CWPS precipitation curve observed for pCRP, the *in vitro* produced mCRP does not follow the general bell-shaped curve typical of aggregation and precipitation of a cross-linked complex (37). The lower level of precipitation and its increased uniformity over a range of CWPS concentrations compared to pCRP, where each pentamer can achieve cross-linking through multi-site CWPS attachment to one or more CWPS, is consistent with the single PCho binding site per monomer reducing the opportunity for aggregation, although modification of the monomer which reduces, but does not abrogate, CWPS binding cannot be discounted. Previous reports using 8 M urea for dissociation have described the production of a monomeric form of CRP which displayed a decrease in solubility [aggregating and forming precipitates at calcium concentrations as low as 0.05 mM (9)] and isoelectric point, and loss of calcium-dependent binding to the prototypical CRP ligand CWPS (20). This suggests that the *in vitro* mCRP produced here at 3 M urea differs from that previously described.

The experimental evidence provided by the *in vitro* dissociation studies, providing a monomeric CRP with MW approximately 23 kDa which reversibly binds PCho in a calcium-dependent manner, precipitates with CWPS and is recognized by an anti-CRP monoclonal antibody, formed the basis for the methodology developed for the analysis of monomeric C-reactive protein in human serum from patients with markedly elevated CRP levels >100 mg/L. Our aim was to carry out this analysis independent of antibodies specific for one or more forms of mCRP, or specific for particular mCRP epitopes, using samples from patients with clinically recorded raised pCRP levels, >100 mg/L. Thus, PCho affinity chromatography followed by size exclusion, complemented by western blotting and ELISA were used for the isolation, purification, and detection of serum monomeric CRP. The average concentration of pentameric CRP in the patient serum samples was calculated from the ELISA data to be 23.3 mg/L, with the minimum value calculated to be 2.5 mg/L and the maximum 71.3 mg/L (Figure 5). The difference between the pCRP levels determined here and those provided by clinical assay, all of which were reported as >100 mg/L, suggests significant losses during the purification procedure and the need for more efficient processes for the quantification of CRP forms.

Monomeric CRP was successfully identified by ELISA in the purified CRP of all the 40 patient serum samples (Figure 5) with three of the four highest concentrations also being detected on western blots. Combined with the PCho affinity chromatography used for purification, our results demonstrate that the serum monomeric CRP not only displays the same ability as the dissociated *in vitro* mCRP to reversibly bind to PCho in a calcium dependent manner, but also is recognized by the same anti-CRP monoclonal as both pCRP and *in vitro* mCRP. From the ELISA, the mean recorded level of monomeric CRP within the serum samples was 1.03 mg/L, with the minimum recorded value being 0.12 mg/L and the maximum recorded value being 3.14 mg/L. Significantly, the two highest mCRP serum concentrations calculated from ELISA (samples 115 and samples 148; Figure 5) were detected by western blotting (Figure 5). There is no correlation between the abundance of the two molecular forms (Figure S7), for example of the ten highest serum pCRP values (Figure 5) only three corresponded to one of the ten highest mCRP values (Figure 5). These results suggest that serum monomeric CRP levels are significantly lower than those for pentameric CRP, with monomeric CRP levels varying across the cohort. Despite the loss of protein in the purification and concentration process, the PCho-binding monomeric CRP is detectable in the serum of patients with markedly elevated CRP levels at an order of magnitude higher levels when compared to previously published data based on serum ELISA (24, 36). While this may be due to the different procedures, to our inclusion of only patients with raised CRP levels >100 mg/L, or to mCRP bound to microparticles evading the PCho affinity extraction (30, 35), it raises the possibility of two different forms of circulating mCRP. As the patient samples used here all have markedly elevated levels of CRP, no conclusions can be drawn from the data presented here as to whether mCRP is present in normal, healthy serum, or the nature of that mCRP if present.

In order to verify that the mCRP identified here in serum is not a product of our purification procedures, commercially sourced native CRP was subjected to those same procedures as the serum samples, involving PCho affinity chromatography (which would isolate any of the calcium dependent PCho binding soluble mCRP in addition to pCRP), concentration, size exclusion, and ELISA using the same antibody (CRP-8). The results (Figure 6) show that mCRP is not a product of our procedures, with no mCRP detectable by ELISA. Our conclusion therefore is that the calcium dependent PC binding, soluble mCRP identified and characterized here is present at detectable levels in serum with markedly elevated levels of CRP.

The mCRP isolated from human serum samples, in this case where pCRP levels were clinically assayed as >100 mg/L, and the 3 M urea for 10 weeks dissociated mCRP samples share key binding properties. Both display the ability to reversibly bind to PCho in a calcium-dependent manner and in common with pCRP both are recognized by the same anti-CRP monoclonal antibody. Precipitation of the urea dissociated mCRP with CWPS further suggests that both forms investigated here retain the overall structural and functional properties of CRP monomers within the pentameric CRP molecule. This suggests that the mCRP detected here in serum with markedly elevated CRP levels is similar to the membrane-bound dissociated subunit with near native structure proposed by Lv and Wang (22), Zhang et al. (36), and Ji et al. (25) rather than the variety of reduced, denatured and refolded mCRP reported elsewhere. It has been suggested that a near-native membrane-bound mCRP is an intermediate step between dissociation, following pCRP membrane attachment, and release following conformational change (25, 27). Whether the serum mCRP detected here is released from the cell surface following pCRP dissociation or whether it originates elsewhere is not clear. If it is of cell surface bound pCRP origin then this suggests that conversion to a conformationally distinct mCRP following dissociation is not a prerequisite for release from the membrane.

The overlapping characteristics of the *in vitro* and *ex vivo* mCRP reported here suggest that a physiologically relevant monomeric CRP, which retains near native pCRP subunit structure and function, has been produced through *in vitro* dissociation of pentameric CRP using 3 M urea over an extended period. This has the potential to provide not only new routes to quantification of both pCRP and mCRP in the clinical setting, but also a firm basis for the in-depth study of the structure, function and diversity of physiological CRP.

## Data Availability Statement

All datasets generated for this study are included in the article/Supplementary Material.

## Ethics Statement

The studies involving human participants were reviewed and approved by UK National Health Service Research Ethics Service (NRES) Committee South Central—Oxford B (REC reference: 15/SC/0179). The patients/participants provided their written informed consent to participate in this study.

## Author Contributions

RW, JM, JL, and HW produced and characterized the *in vitro* mCRP. RW isolated and characterized the serum mCRP. AF managed and oversaw the collection of serum samples. AS conceived and managed the programme of work and led the interpretation of the results with input from TG, JL, and HW. TG carried out revisions of the manuscript drafted by RW and AS and completed production of the final version including the figures. All authors contributed to refinement of the manuscript and all approved the final version to be published.

### Conflict of Interest

The authors declare that the research was conducted in the absence of any commercial or financial relationships that could be construed as a potential conflict of interest.

## Abbreviations

CRP: human C-reactive protein
pCRP: pentameric human C-reactive protein
mCRP: monomeric human C-reactive protein
BSA: bovine serum albumin
PBS: phosphate buffered saline
TMB, 3, 3′, 5, 5′: tetramethylbenzidine
APC: *p-*aminophenyl phosphoryl choline
FPLC: Fast Protein Liquid Chromatography
CWPS: *Streptococcus pneumoniae* cell wall C-polysaccharide
NRES: National Health Service Research Ethics Service
EDTA: ethylenediaminetetraacetic acid
ELISA: enzyme linked immunosorbent assay
tris: tris(hydroxymethyl)aminomethane
PCho: phosphocholine.
