# Supplementary material for: Monomeric C-Reactive Protein in Serum With Markedly Elevated CRP Levels Shares Common Calcium-Dependent Ligand Binding Properties With an in vitro Dissociated Form of C-Reactive Protein
Source: Front Immunol. 2020 Feb 4;11:115. doi: 10.3389/fimmu.2020.00115 (PMC7010908; doi:10.3389/fimmu.2020.00115)

## Slide 1
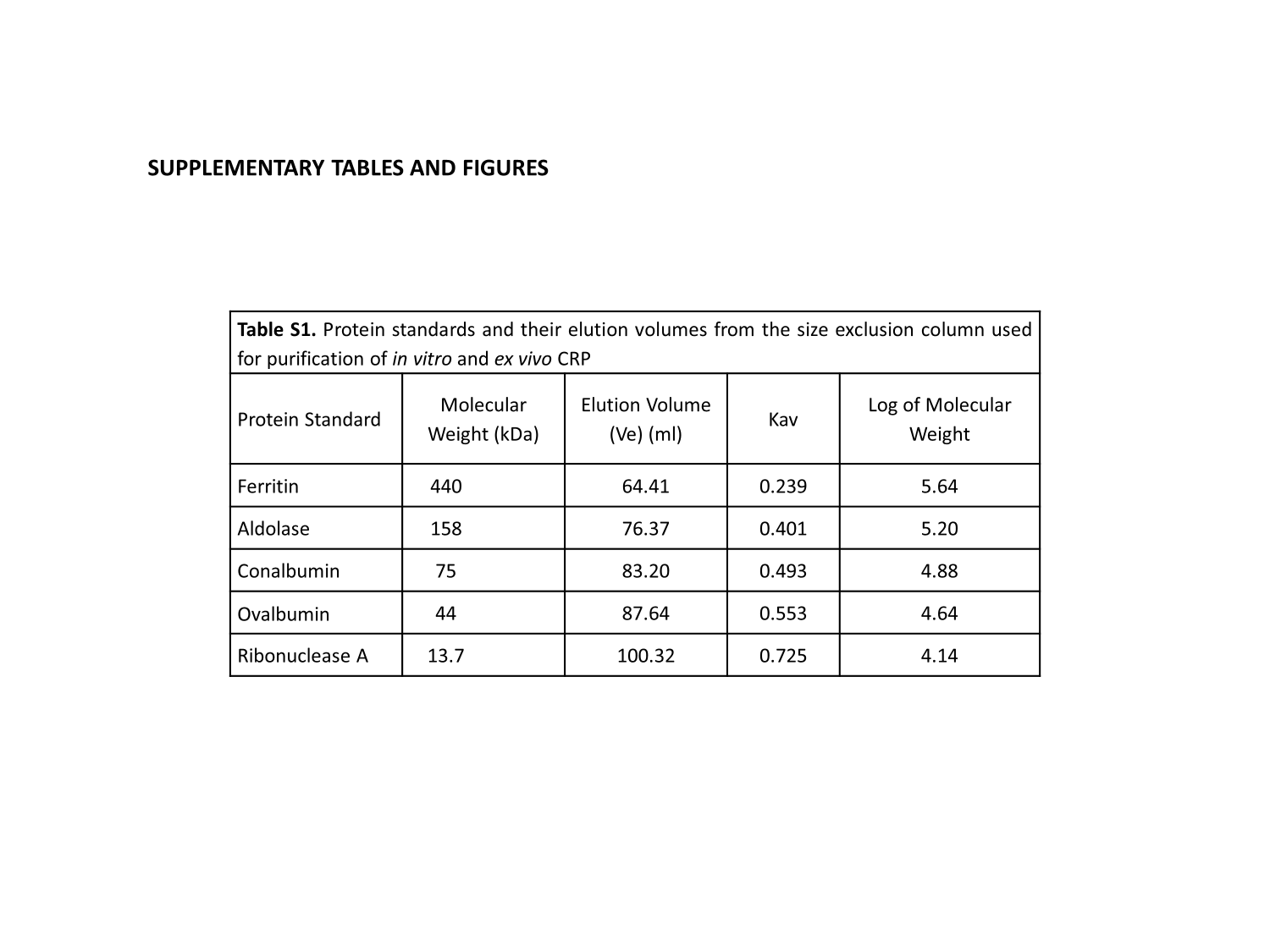

## Slide 2
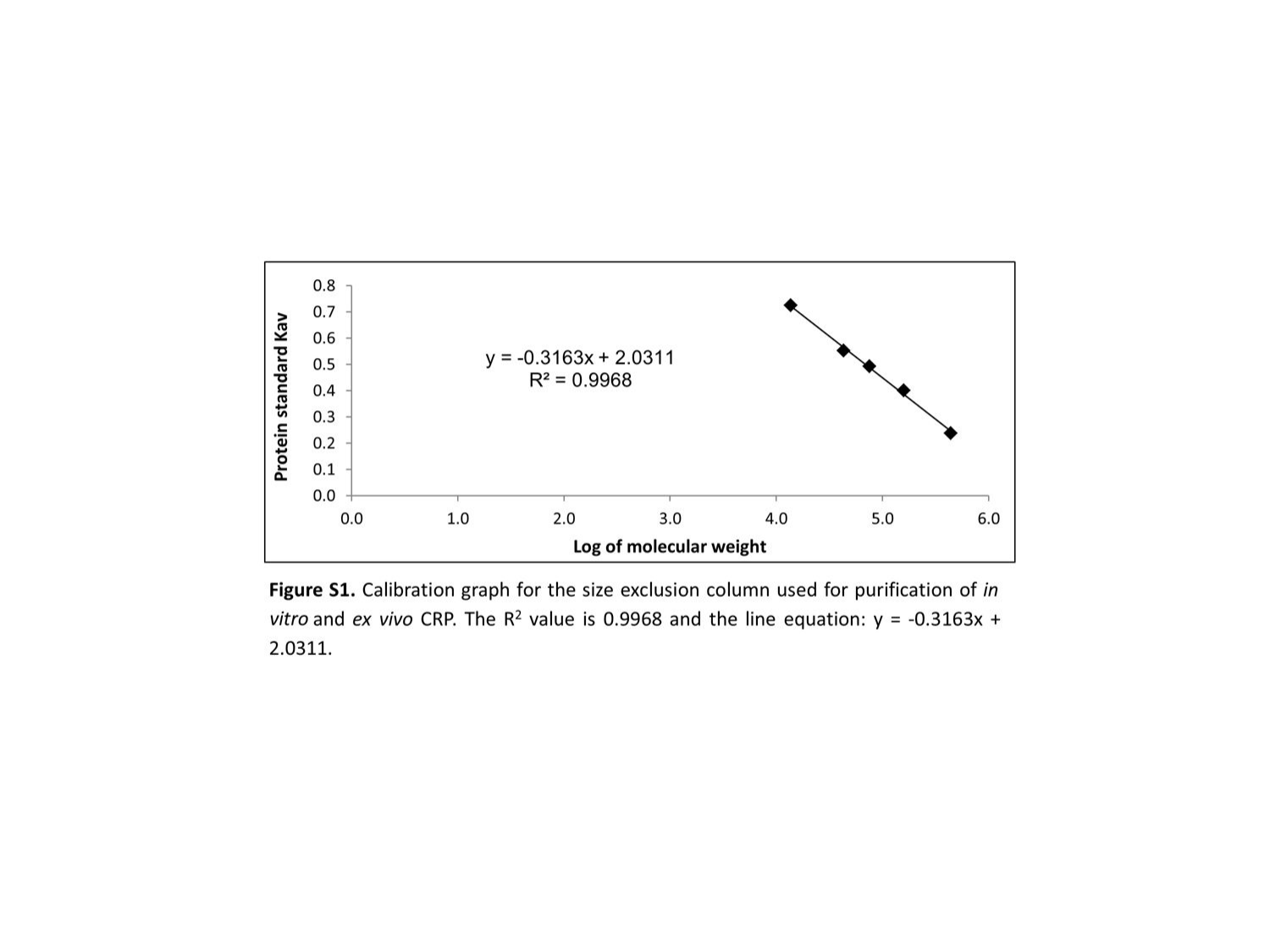

## Slide 3
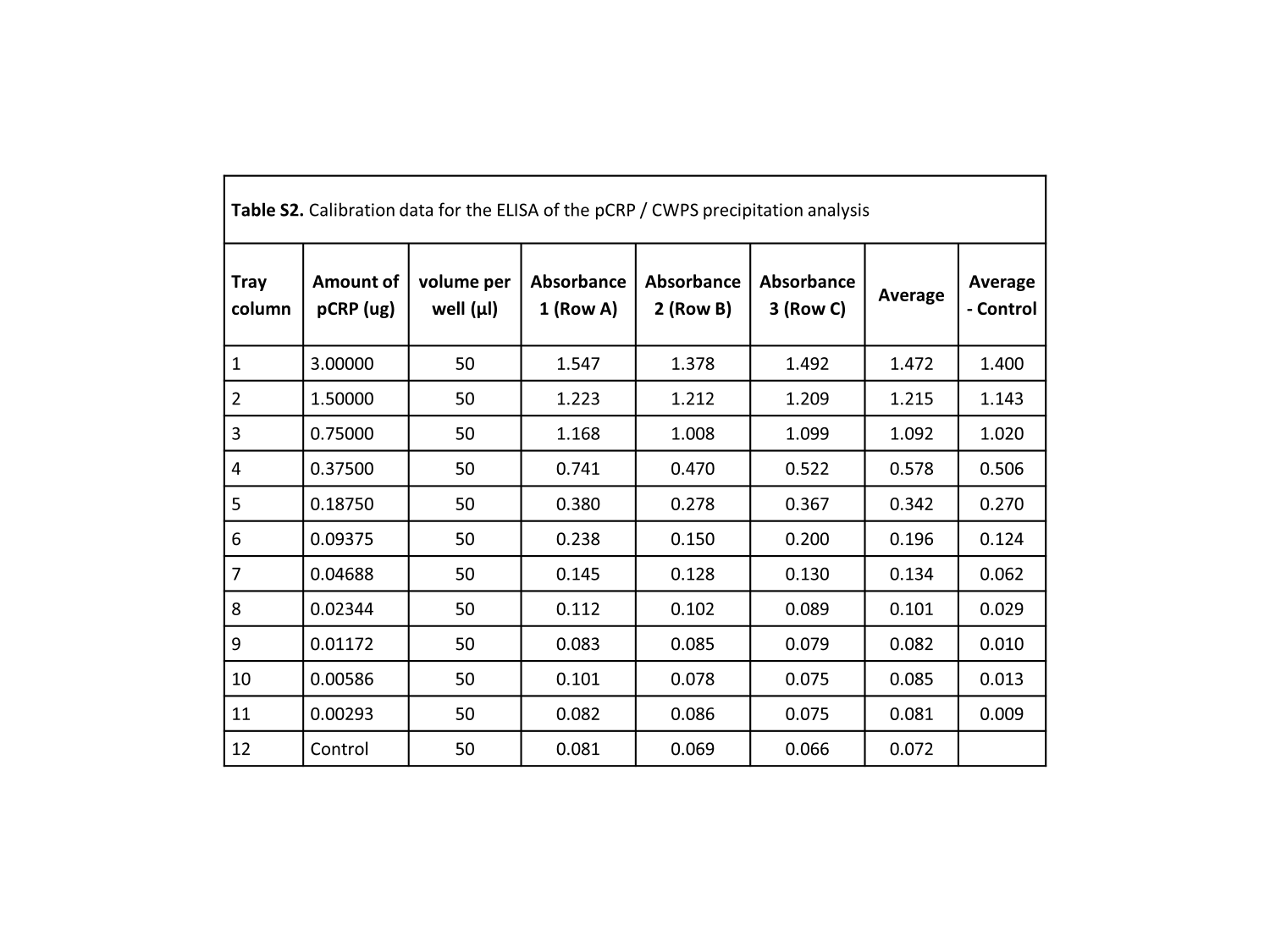

## Slide 4
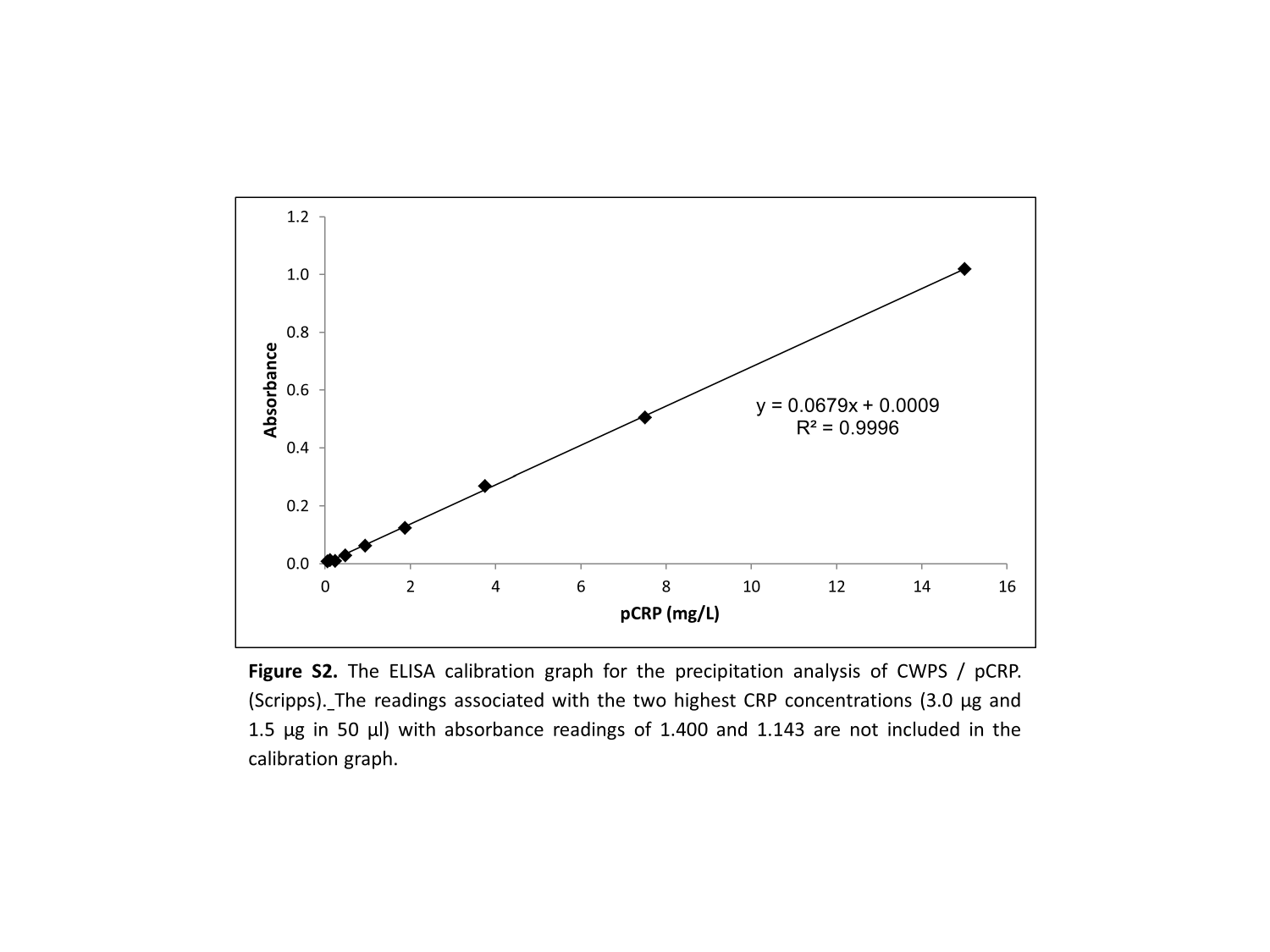

## Slide 5
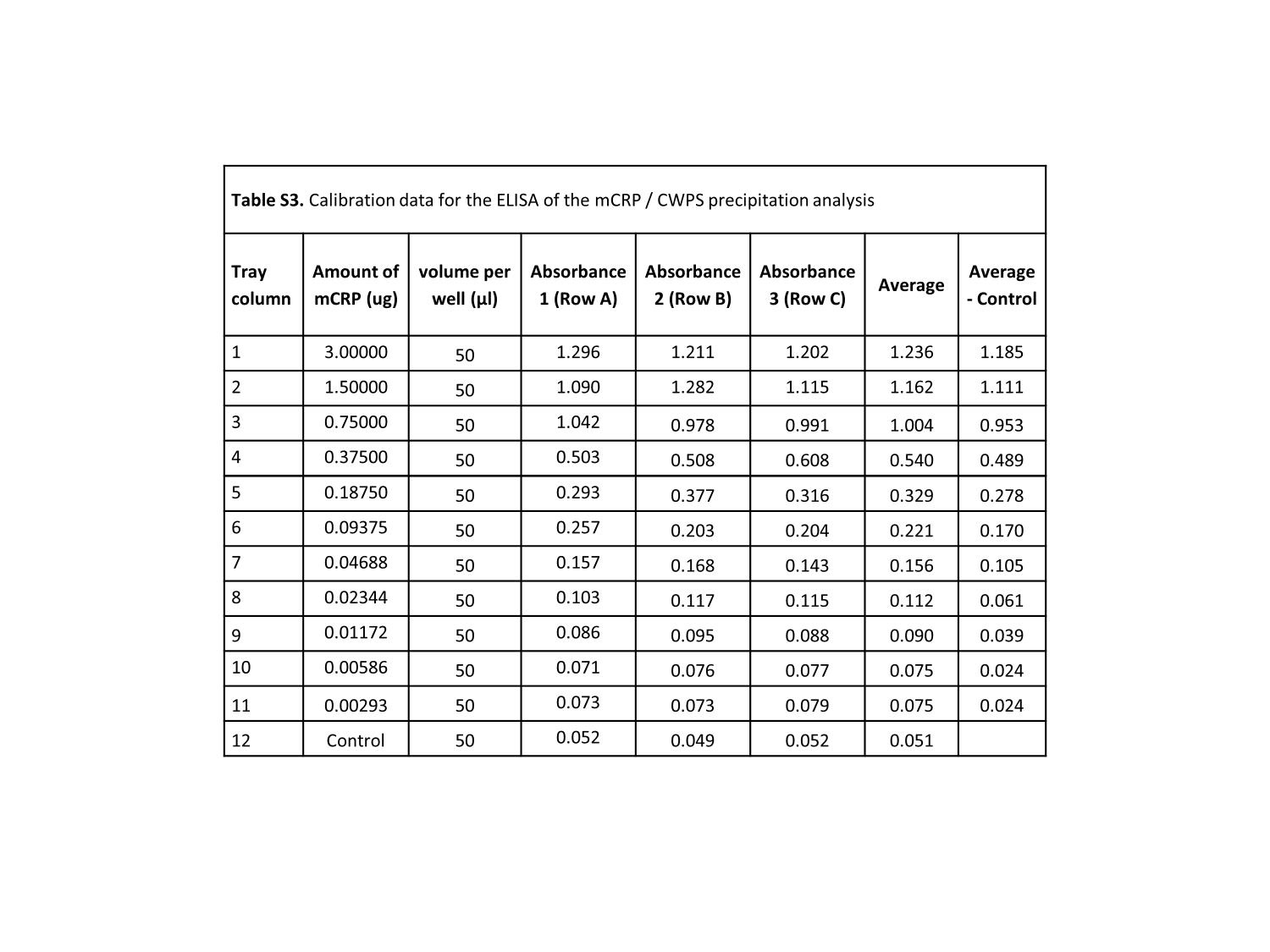

## Slide 6
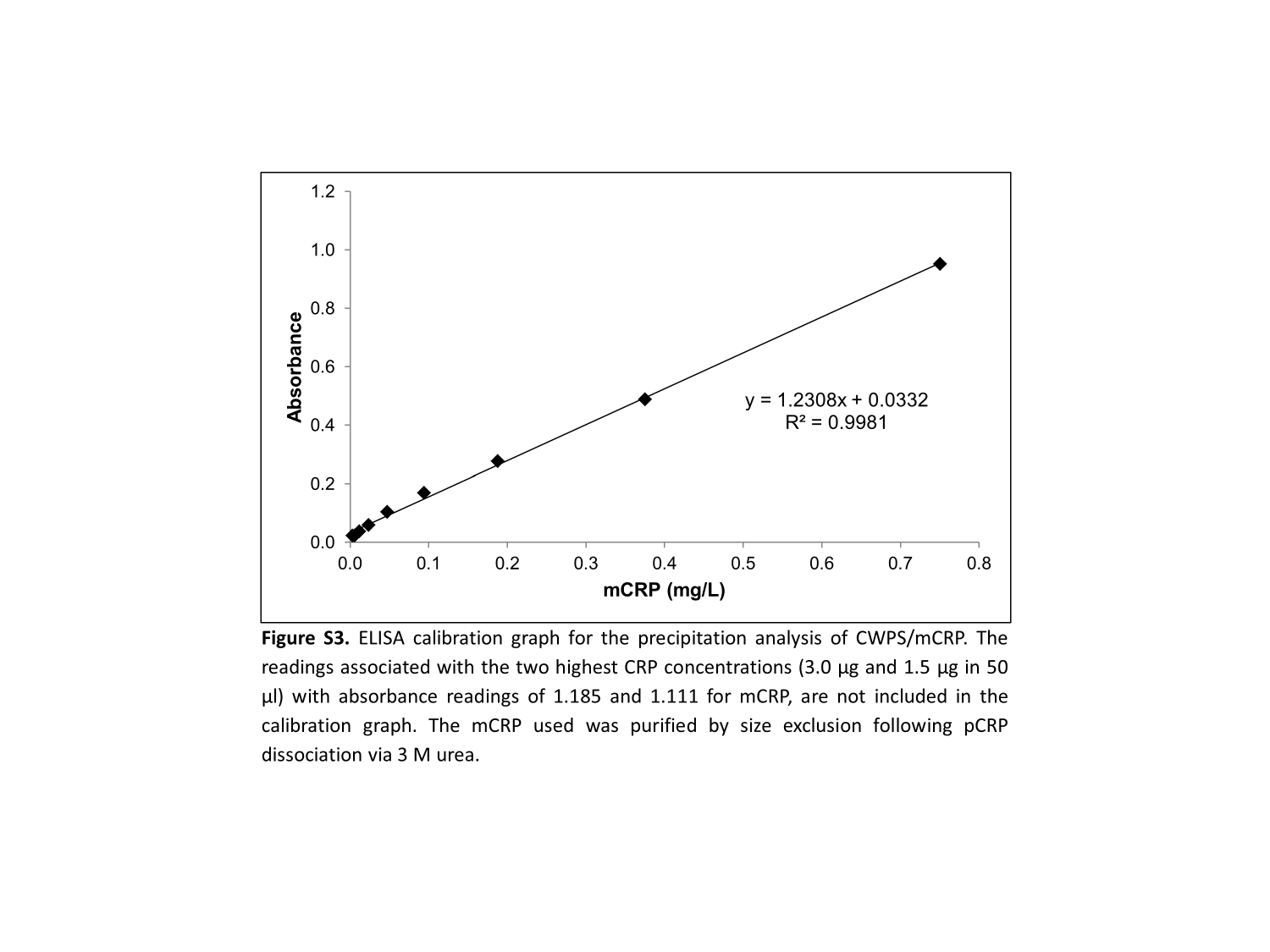

## Slide 7
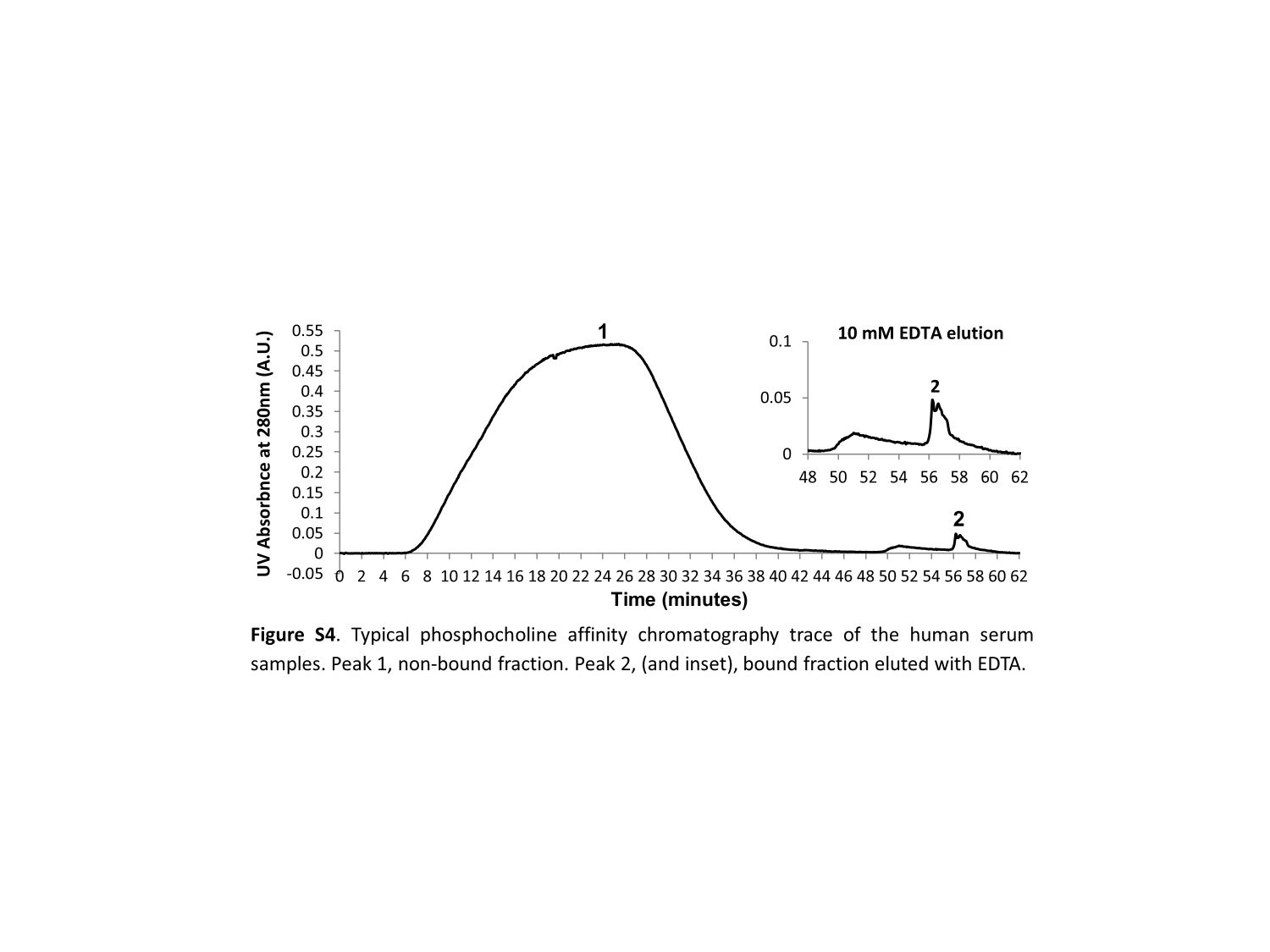

## Slide 8
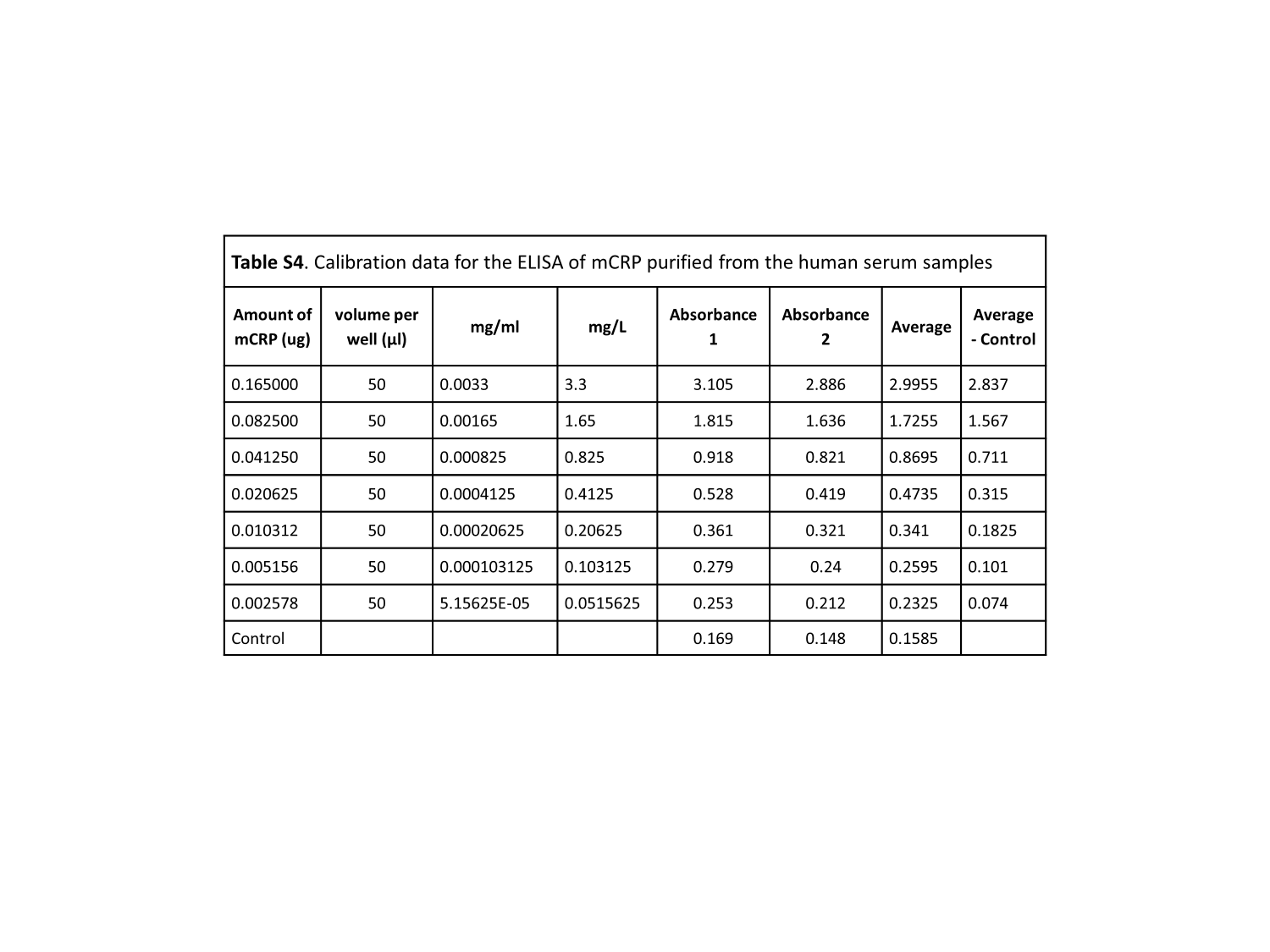

## Slide 9
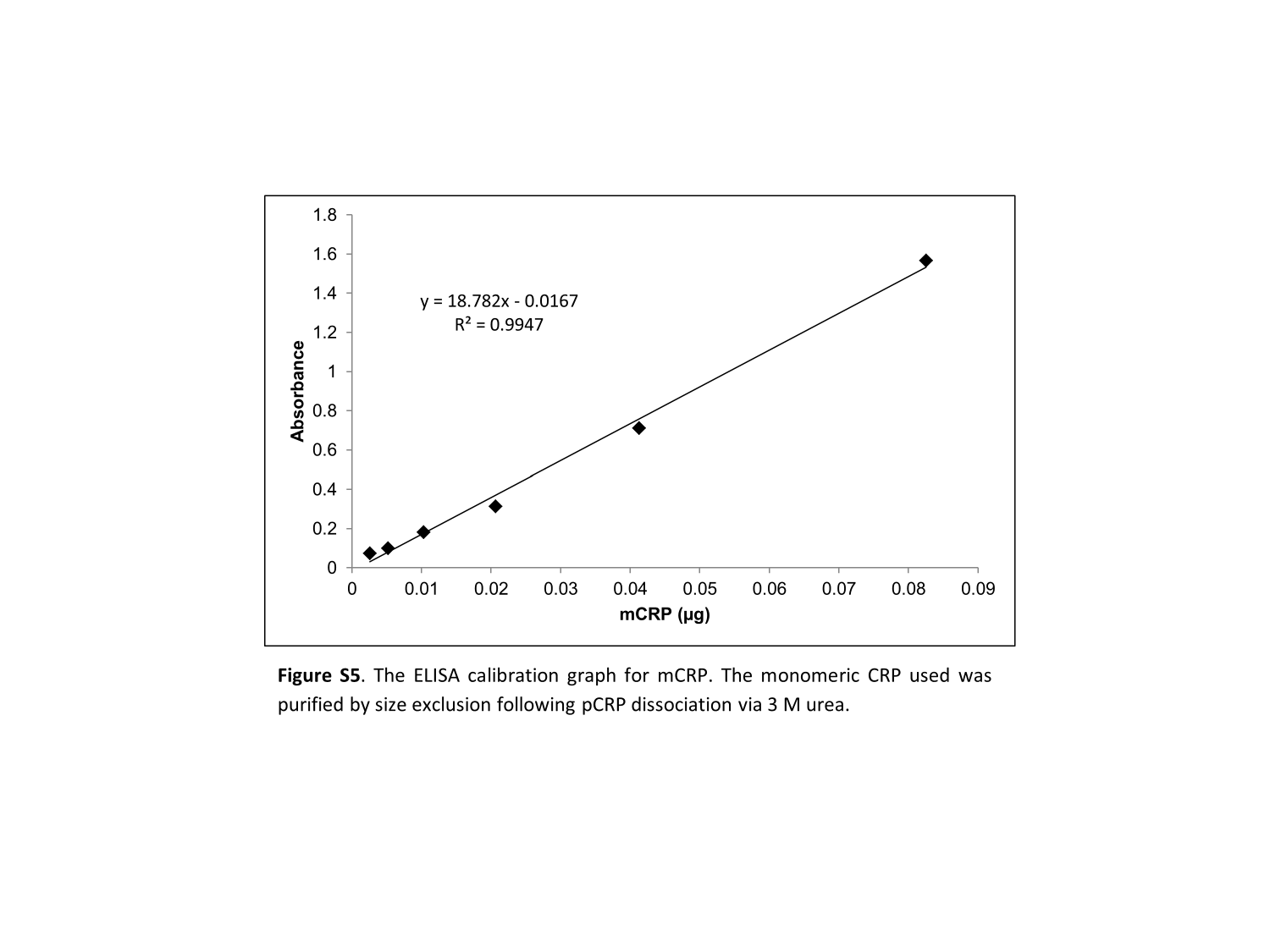

## Slide 10
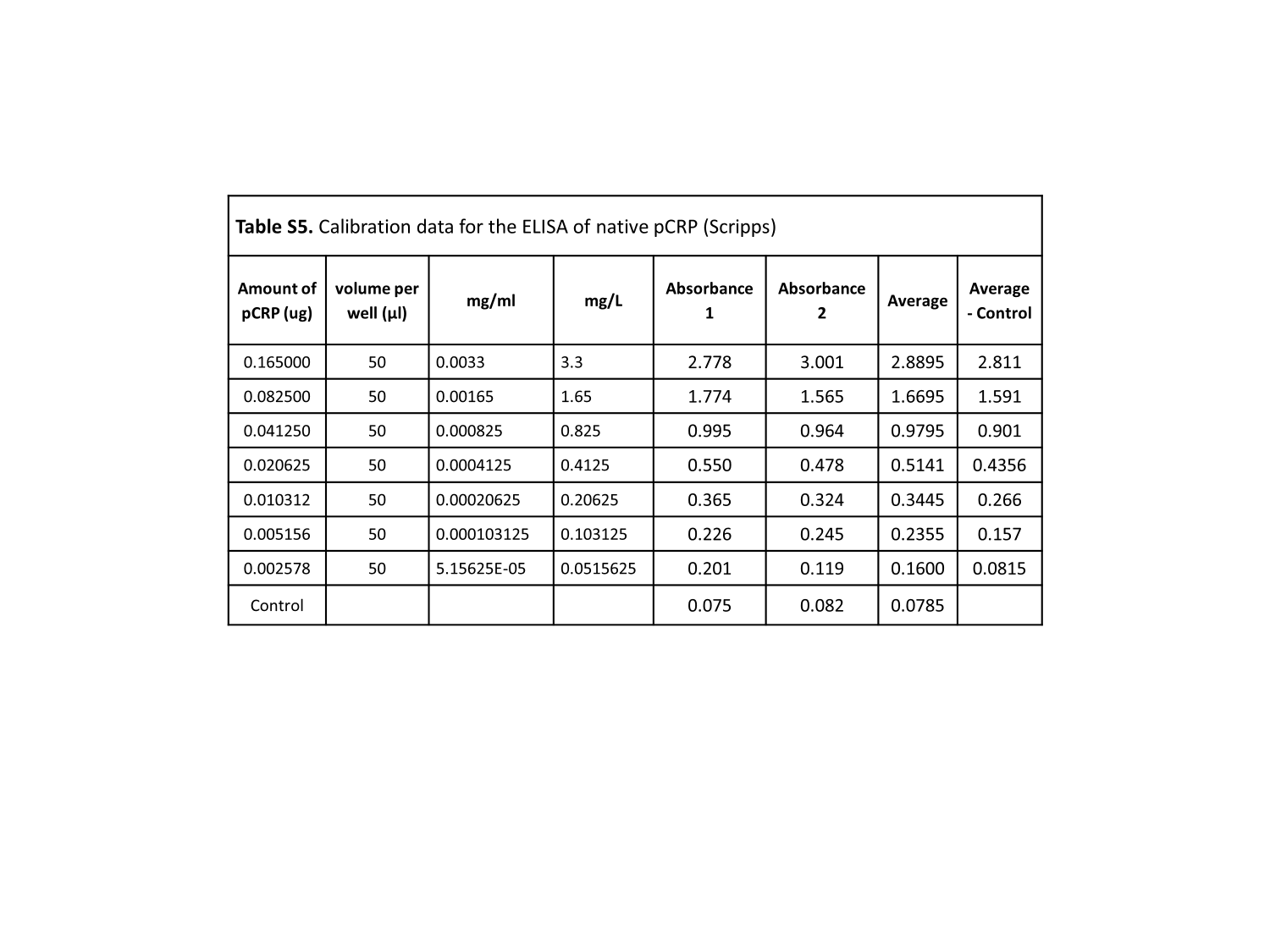

## Slide 11
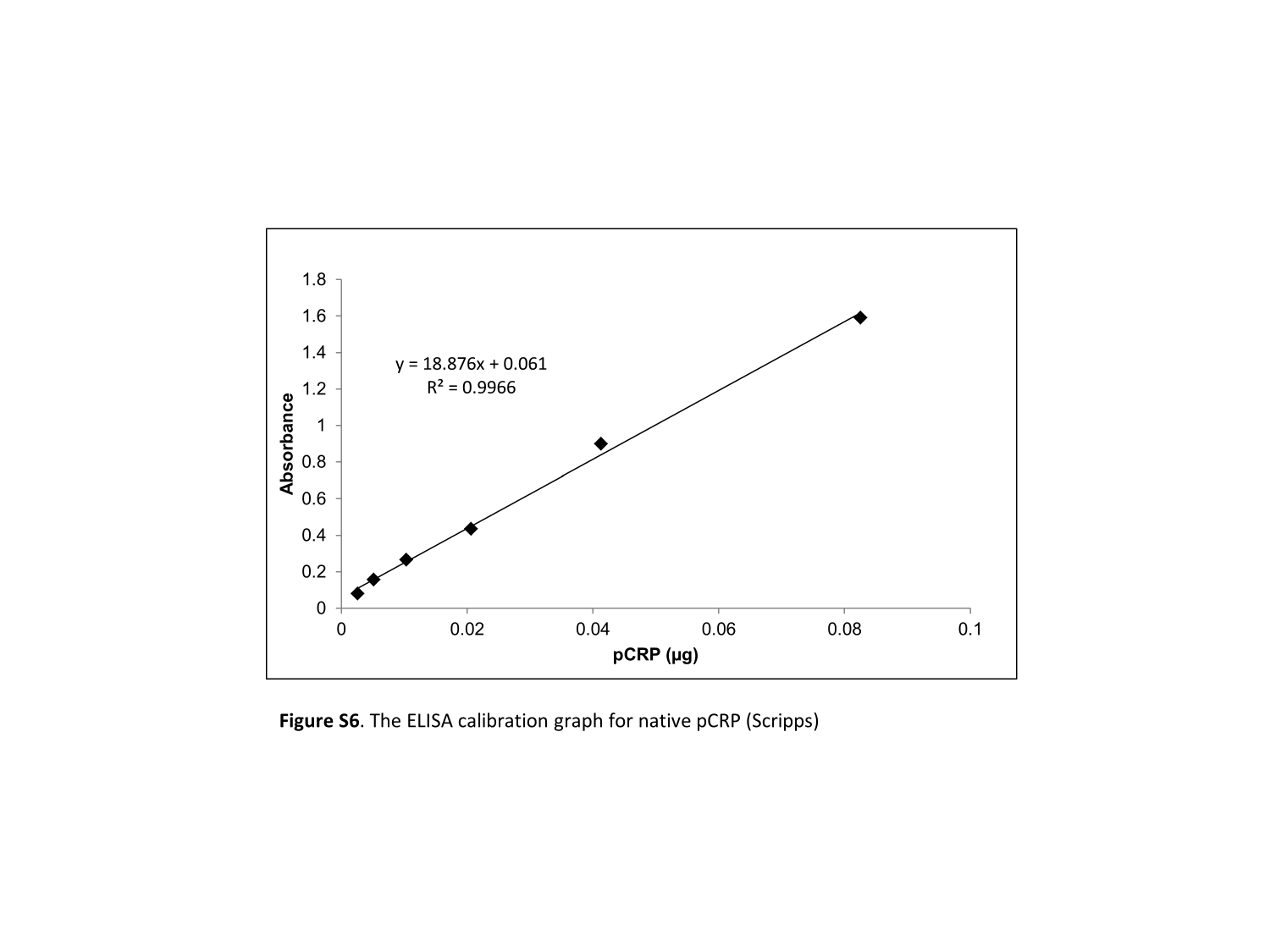

## Slide 12
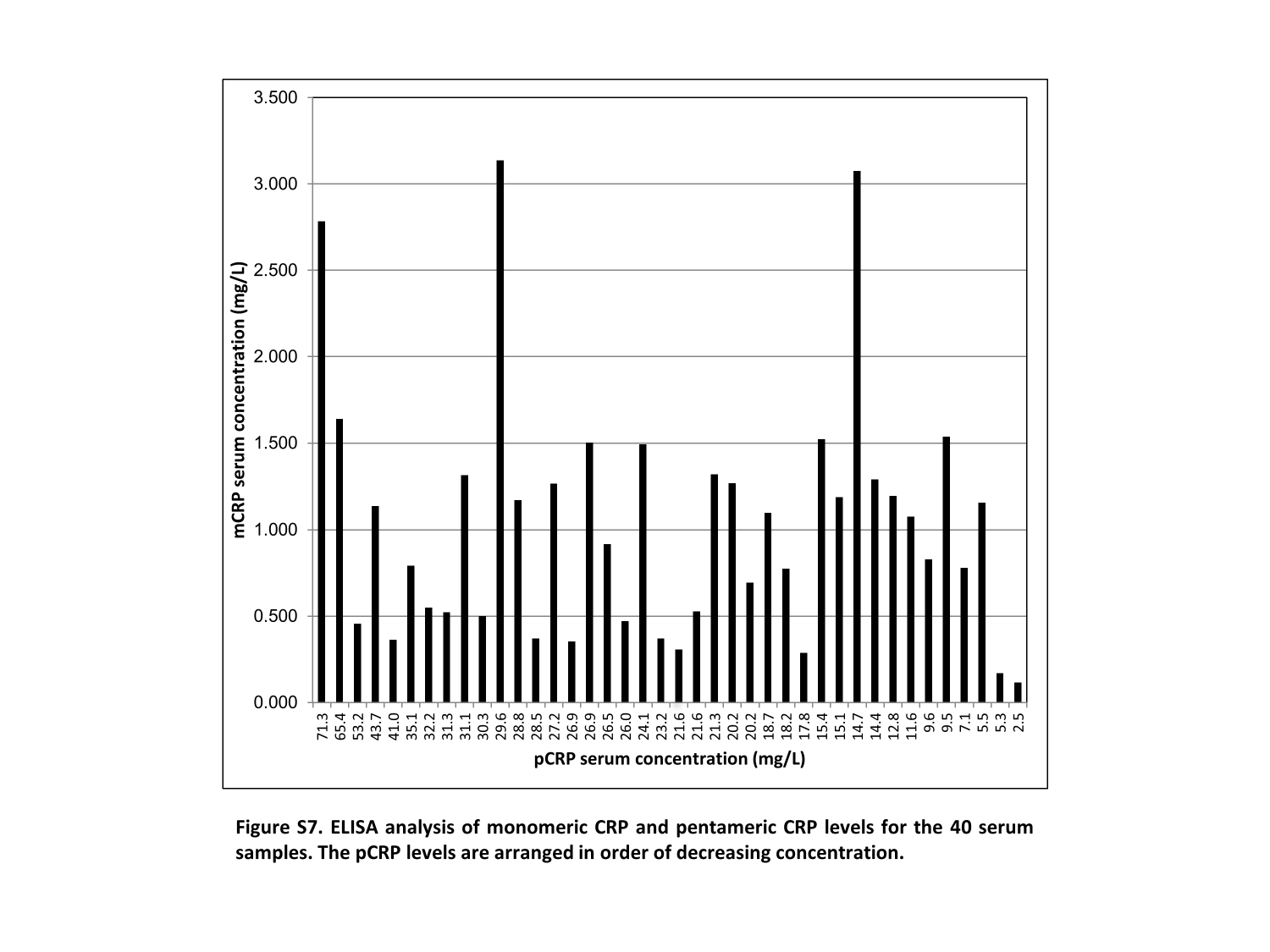

## Slide 13
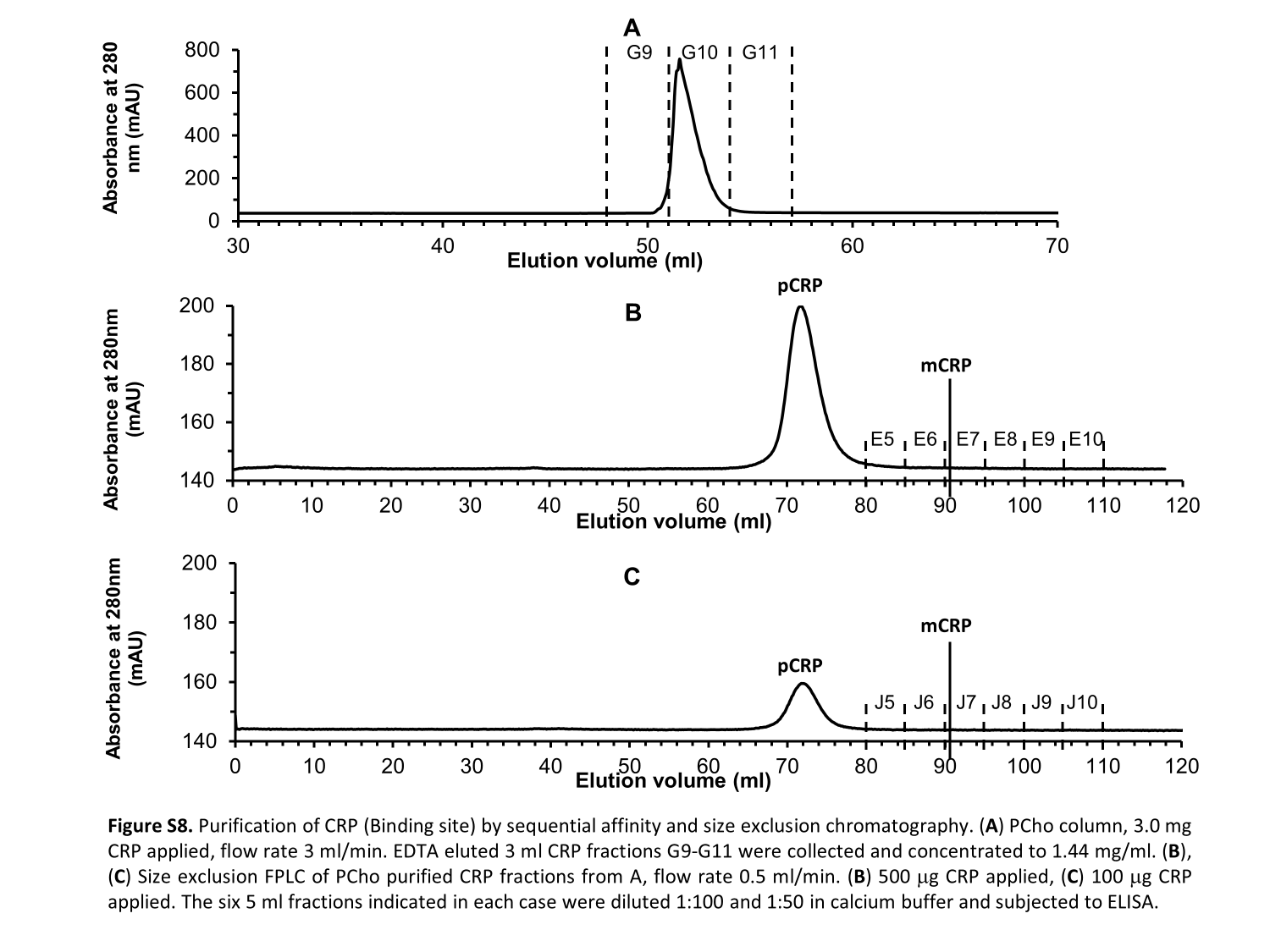

## Slide 14
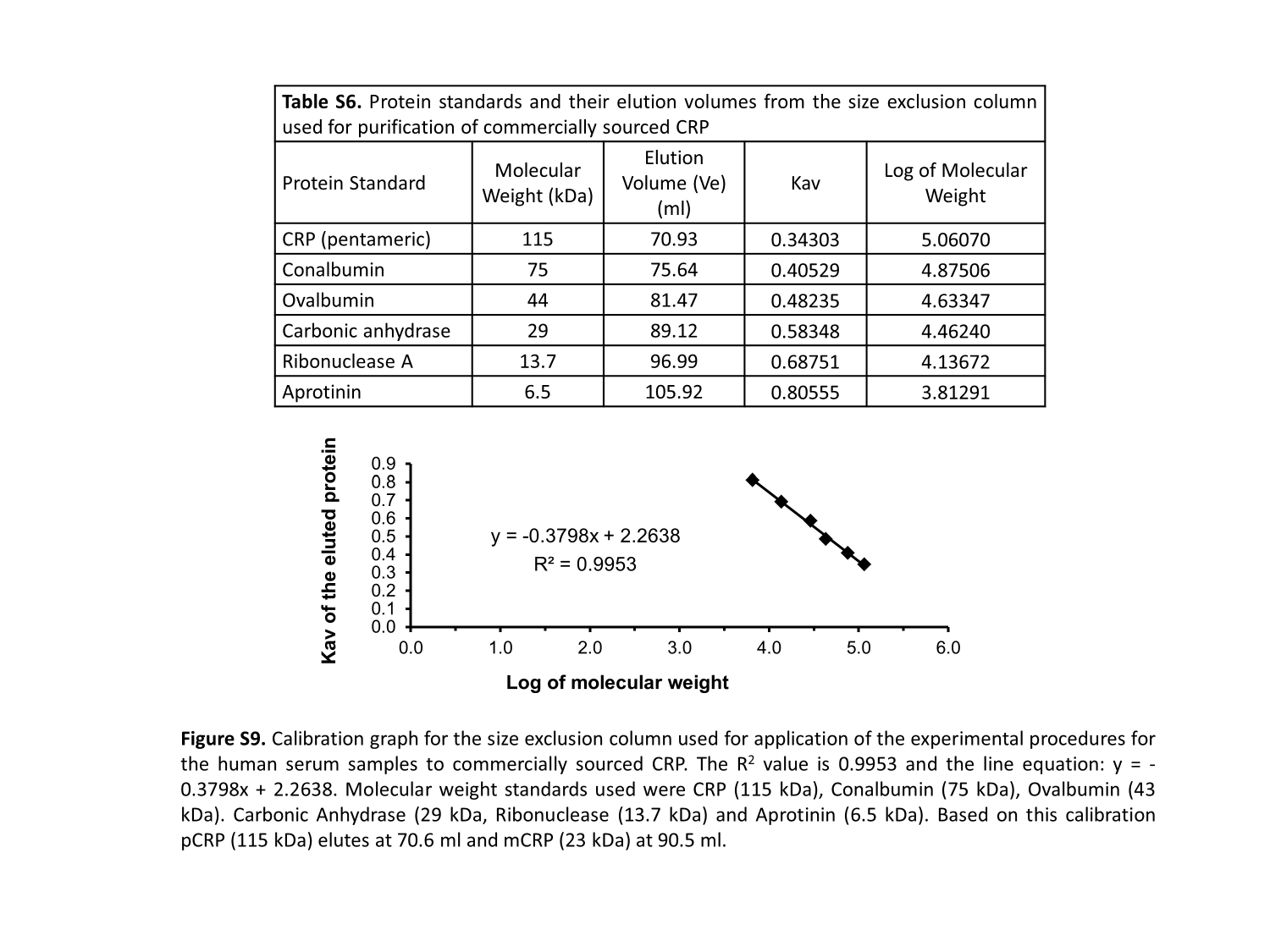

## Slide 15
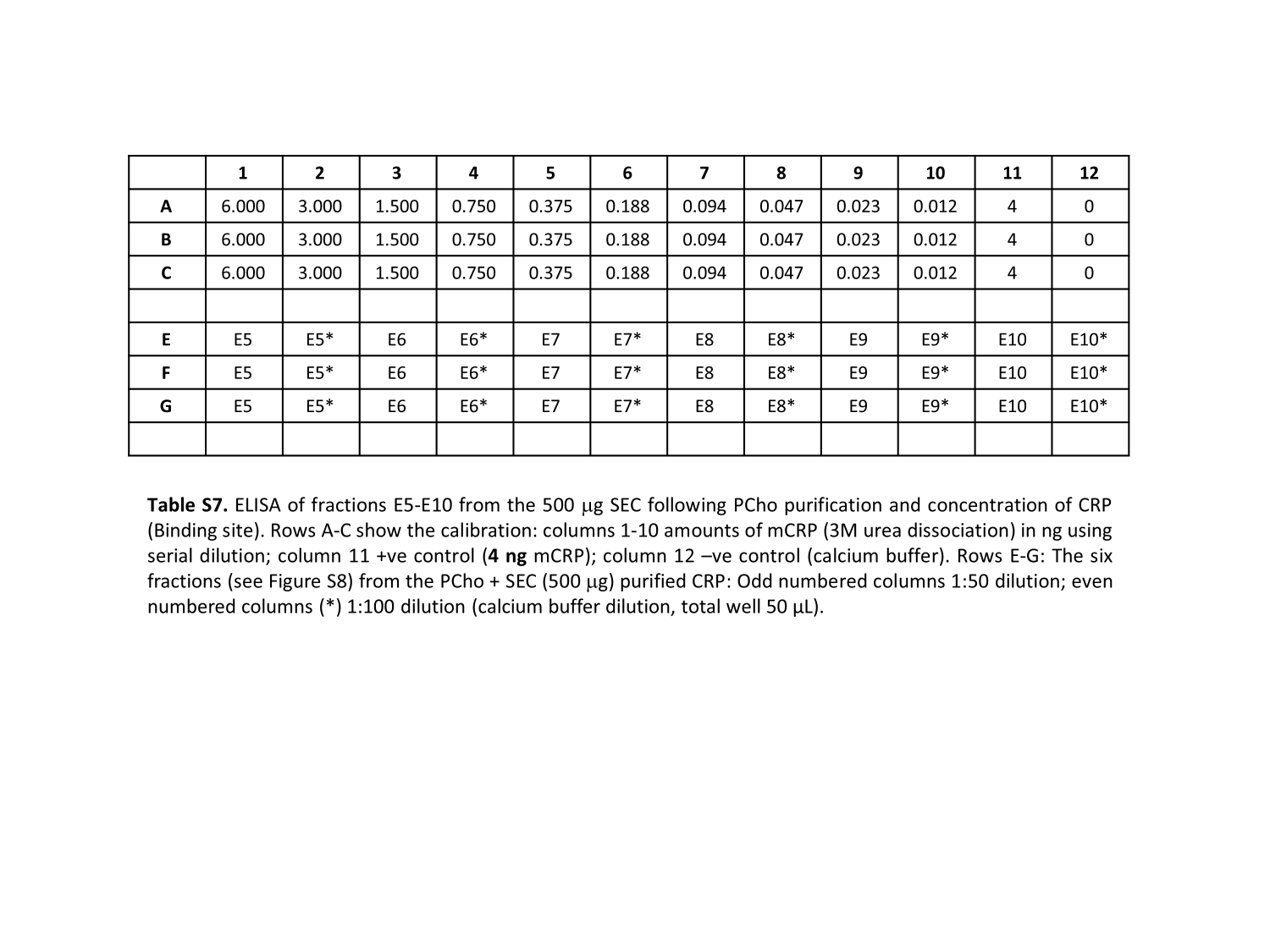

## Slide 16
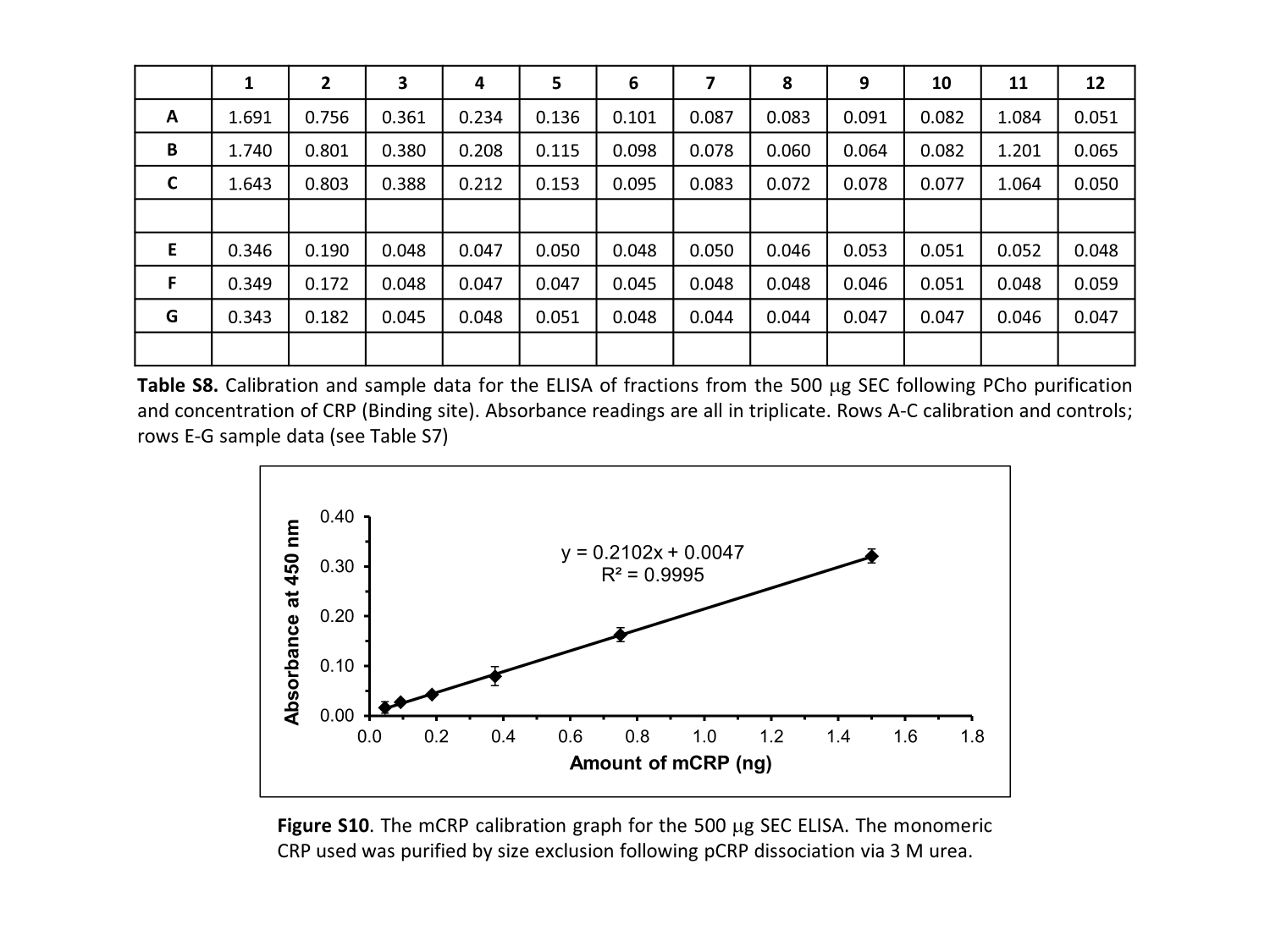

## Slide 17
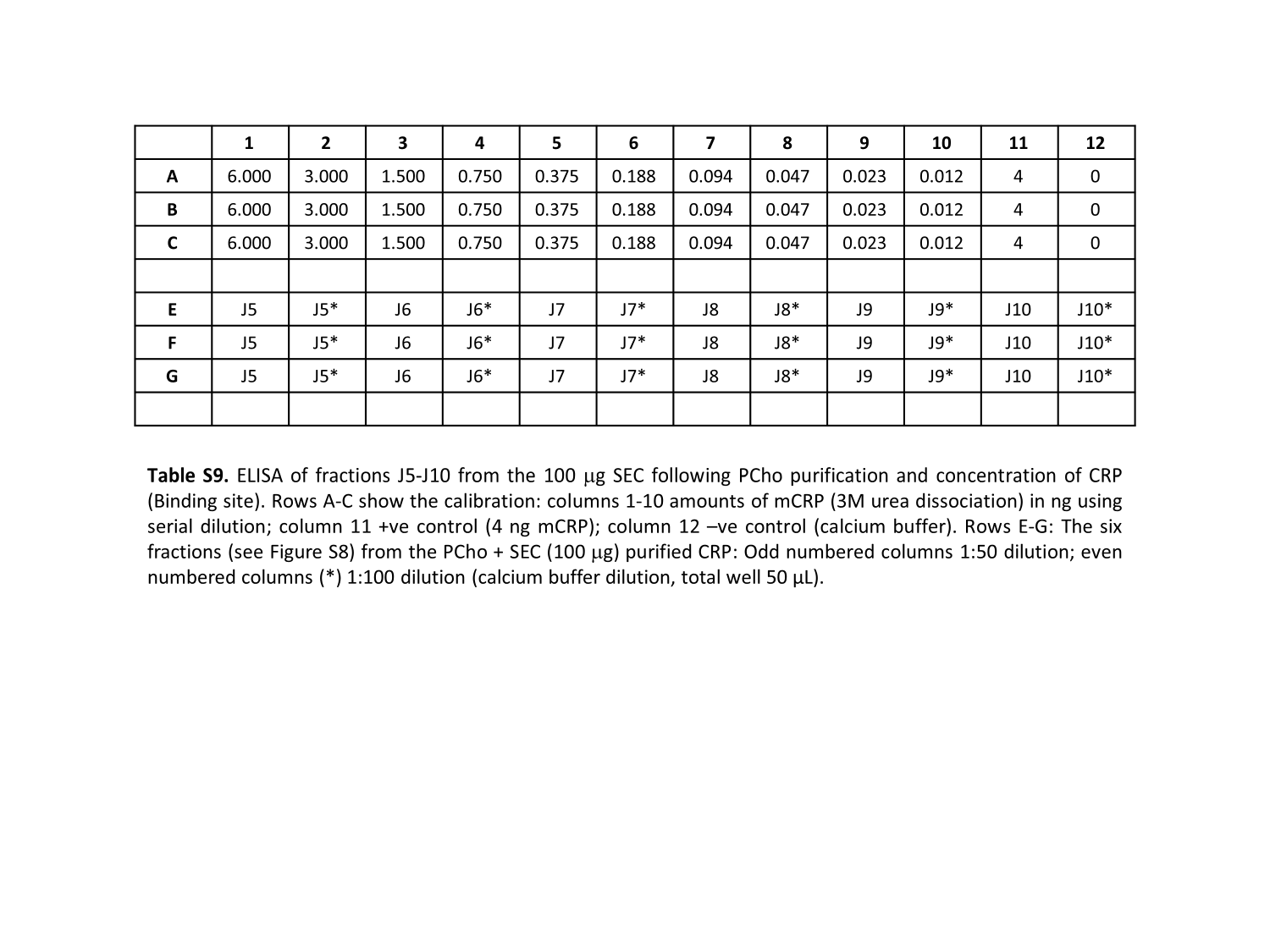

## Slide 18
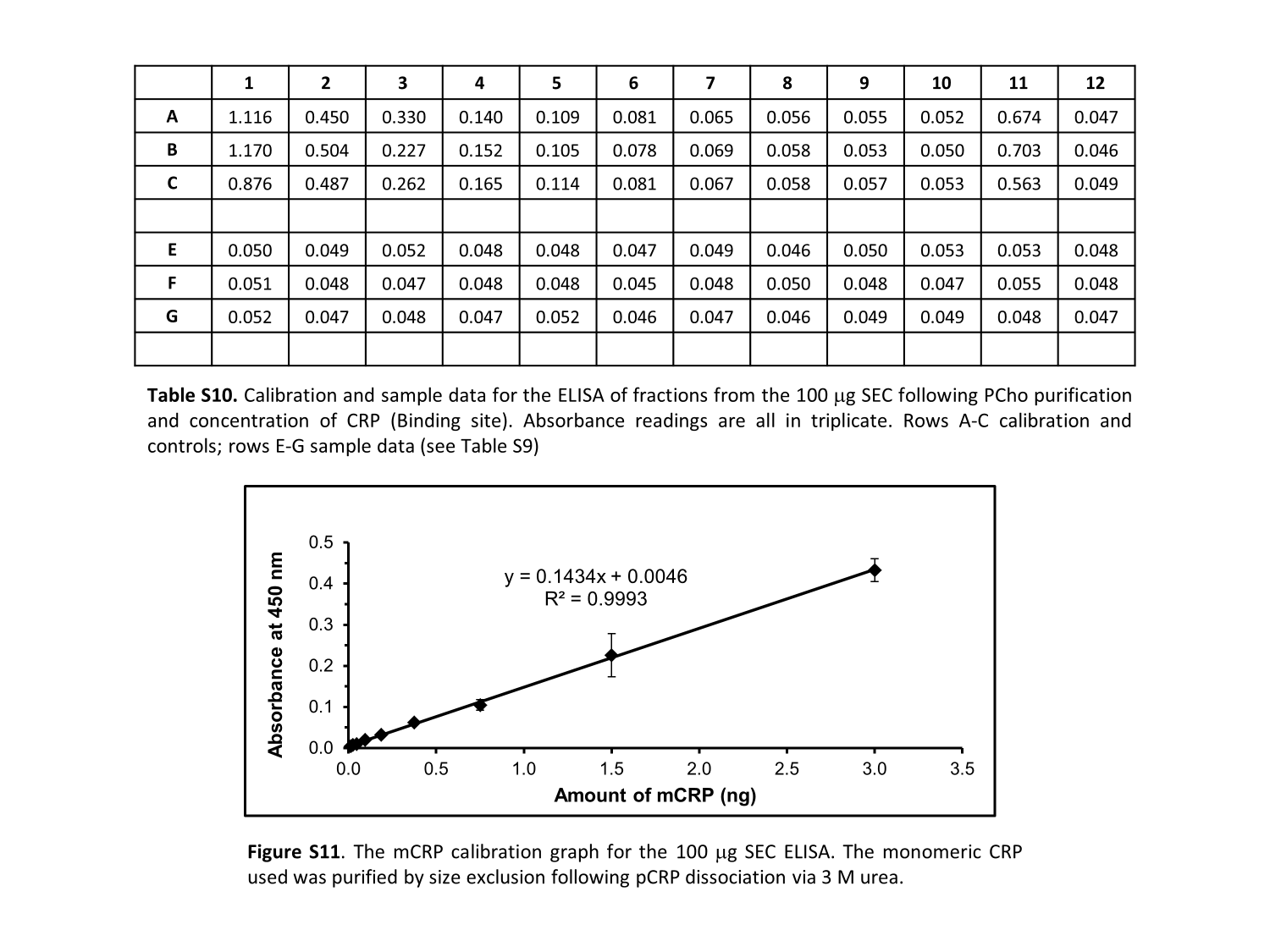

Supplement: Supplementary file 1 [file Presentation_1.PPTX]
